# Computer simulation of the SARS-CoV-2 contamination risk in a large dental clinic

**DOI:** 10.1063/5.0043934

**Published:** 2021-03-29

**Authors:** Jonathan Komperda, Ahmad Peyvan, Dongru Li, Babak Kashir, Alexander L. Yarin, Constantine M. Megaridis, Parisa Mirbod, Igor Paprotny, Lyndon F. Cooper, Susan Rowan, Clark Stanford, Farzad Mashayek

**Affiliations:** 1 Department of Mechanical and Industrial Engineering, University of Illinois at Chicago, Chicago, Illinois 60607, USA; 2 Department of Electrical and Computer Engineering, University of Illinois at Chicago, Chicago, Illinois 60607, USA; 3 College of Dentistry, University of Illinois at Chicago, Chicago, Illinois 60612, USA

## Abstract

COVID-19, caused by the SARS-CoV-2 (severe acute respiratory syndrome coronavirus 2) virus, has been rapidly spreading worldwide since December 2019, causing a public health crisis. Recent studies showed SARS-CoV-2's ability to infect humans via airborne routes. These motivated the study of aerosol and airborne droplet transmission in a variety of settings. This study performs a large-scale numerical simulation of a real-world dentistry clinic that contains aerosol-generating procedures. The simulation tracks the dispersion of evaporating droplets emitted during ultrasonic dental scaling procedures. The simulation considers 25 patient treatment cubicles in an open plan dentistry clinic. The droplets are modeled as having a volatile (evaporating) and nonvolatile fraction composed of virions, saliva, and impurities from the irrigant water supply. The simulated clinic's boundary and flow conditions are validated against experimental measurements of the real clinic. The results evaluate the behavior of large droplets and aerosols. We investigate droplet residence time and travel distance for different droplet diameters, surface contamination due to droplet settling and deposition, airborne aerosol mass concentration, and the quantity of droplets that escape through ventilation. The simulation results raise concerns due to the aerosols' long residence times (averaging up to 7.31 min) and travel distances (averaging up to 24.45 m) that exceed social distancing guidelines. Finally, the results show that contamination extends beyond the immediate patient treatment areas, requiring additional surface disinfection in the clinic. The results presented in this research may be used to establish safer dental clinic operating procedures, especially if paired with future supplementary material concerning the aerosol viral load generated by ultrasonic scaling and the viral load thresholds required to infect humans.

## I. INTRODUCTION

Novel COVID-19 was first reported in Wuhan, China in December 2019 (Huang *et al.*, 2020) and rapidly spread around the world over the following months (World Health Organization, 2020a; 2020b). This outbreak was declared a pandemic by the World Health Organization (WHO) on March 11, 2020. The novel coronavirus causing the COVID-19 disease is named SARS-CoV-2 (severe acute respiratory syndrome coronavirus 2), which belongs to the pathogen family that was responsible for another respiratory illness (SARS-CoV-1) outbreak in 2002–2003.

Respiratory viruses can be transmitted from humans to humans through several plausible pathways, such as direct and indirect contact, large respiratory droplets, and aerosols (Shiu *et al.*, 2019). Direct transmission refers to contact with the patient, whereas indirect transmission occurs from a reservoir, such as a contaminated surface or object. Respiratory droplet and aerosol transmission imply viral shedding from humans through virus-bearing particles expelled via coughing/sneezing and even from normal breathing/talking by the infected person (Leung *et al.*, 2020; Shiu *et al.*, 2019). These viral shedding mechanisms produce droplets of various sizes, including large droplets and aerosols (Kutter and Spronken, 2018). The currently accepted threshold for droplets to be considered aerosols is 100 *μ*m (Prather *et al.*, 2020). This recently revised definition of an aerosol more accurately accounts for the differing aerodynamic behavior of larger and smaller virion-bearing droplets and alters the historical definitions of 5 *μ*m (Gralton *et al.*, 2011; Fennelly, 2020). Additionally, there is a clear distinction between droplets and droplet nuclei, which do not continue to evaporate. Droplet or aerosol nuclei are capable of wider-spread since they do not fully evaporate and remain airborne for prolonged periods of time (Duguid, 1946; Verma *et al.*, 2020). Airborne transmission through droplets and aerosols facilitates the spread of viruses among humans and causes outbreaks. Large droplets (over 100 *μ*m) produced as a result of coughing, sneezing, or talking typically travel distances of less than 2 m before settling on surrounding surfaces (Prather *et al.*, 2020). On the other hand, aerosols can remain airborne for hours and can travel farther (Pica and Bouvier, 2012; Fernstrom and Goldblatt, 2013; and Prather *et al.*, 2020).

The early reports from WHO imply that transmission routes of SARS-CoV-2 are mainly from direct contact and large droplets (World Health Organization, 2020a; 2020b). This statement was challenged by the scientists' findings that airborne transmission via aerosols is the primary contagion method of COVID-19 disease (Morawska and Milton, 2020) and altered the WHO interpretation of transmission routes (World Health Organization, 2020a; 2020b). Aerosols remain airborne for hours (or days) and can travel several meters (Morawska and Milton, 2020). The SARS-CoV-2 virus remains viable in aerosols and possesses a potential inhalation exposure to the individuals present in the environment. Several scientific reports support this exposure risk, including a study that has shown that a 5 *μ*m droplet at the height of 1.5 m with typical indoor velocity (less than 0.2 m/s) will traverse tens of meters until settling on a surface (Matthews *et al.*, 1989; Morawska and Milton, 2020). For instance, inhalation of respiratory aerosols caused superspreading of COVID-19 at a regular weekly rehearsal of the Skagit Valley Corale on March 10, 2020 (Miller *et al.*, 2021). Between 32 (confirmed) and 52 (confirmed and suspected) of the 61 people in attendance became infected at the rehearsal despite additional precautions, such as increased distancing and physical contact restrictions. Field experiments established the presence of SARS-CoV-2 RNA in aerosols in Wuhan's hospitals (Liu *et al.*, 2020). Liu *et al.* found that concentrations of SARS-CoV-2 RNA were low in isolation and ventilated patient rooms, but high in toilet areas used by patients. Although they were unable to establish the infection risk in these areas, they determined that SARS-CoV-2 has the potential to be transmitted via aerosols in medical settings. Birgand *et al.*'s (Birgand *et al.*, 2020) more recent assessment of SARS-CoV-2 contamination in hospital settings analyzed 893 air samples across 24 studies and found high positivity rates of SARS-CoV-2 RNA in intensive care units, bathrooms, public areas, and hallways. The study concluded that although viral contamination rates were high, the air was rarely contaminated with viable viruses. Furthermore, tests performed on outdoor particulate matter (PM_10_) in northern Italy identified the presence of SARS-CoV-2 RNA (Setti *et al.*, 2020). This study presented the first evidence that PM_10_ is capable of harboring virions and can be used as an indicator of epidemic recurrence. Another study showed that the SARS-CoV-2 virus remains viable in aerosols with a reduction in infectious titer after 3 h of being airborne (van Doremalen *et al.*, 2020). Most recently, experiments by Lednicky *et al.* (2020) isolated viable SARS-CoV-2 virus from air samples collected in a hospital room occupied by two COVID-19 patients. These results confirm the ability of aerosols to transport viable viruses at distances of 2 m–4.8 m in the absence of aerosol-generating procedures.

Behaviors such as self-quarantining, social distancing, and hand sanitizing minimize contact transmission of SARS-CoV-2. Airborne transmission can be reduced by the use of face-coverings, which prohibit exhalation and inhalation of virus-bearing aerosols (Leung *et al.*, 2020; Dbouk and Drikakis, 2020b; Kumar and Lee, 2020; and Akhtar *et al.*, 2020). However, face-coverings, such as masks, alone do not completely eliminate the risk of infection due to the possibility of leakage (Verma *et al.*, 2020). The combination of social distancing and facial coverings is necessary to minimize infection risk due to aerosols, especially in confined spaces and face-to-face human interactions (Akhtar *et al.*, 2020). The results of Somsen *et al.* (2020) identified small, enclosed spaces, such as restrooms or elevators, and public spaces with low ventilation as having increased aerosol exposure risks. Their results also highlighted the importance of adequate indoor ventilation to reduce aerosol persistence times. The recent study of Chaudhuri *et al.* (2020) used an *ab initio* disease spread model to determine that aerosols with initial diameters ranging from 10 *μ*m to 50 *μ*m pose the highest infection probability in quiescent indoor air-conditioned spaces. The concerns of these results are compounded when considering that even small droplets under 20 *μ*m can leave residues after evaporation, causing viral spread through surface contamination (He *et al.*, 2021).

There have been several recent numerical studies focusing on the risks associated with virus-laden droplets and aerosols. Recently, Dbouk and Drikakis (2020a) simulated the transport, dispersion, and evaporation of human saliva droplets in varying wind speeds and found that 2 m social distancing may not be sufficient under certain circumstances. Li *et al.* (2020b) performed a similar study simulating an outdoor tropical environment while varying both relative humidity and wind speed. Their results agreed with Dbouk and Drikakis, and highlighted the importance of humidity. Another study investigated the dispersion of cough droplets in the wake of a walking person and found concentrations at low heights behind the individuals (Li *et al.*, 2020d). Pendar and Pascoa (Pendar and Pascoa, 2020) simulated sneezing and coughing in an enclosed environment with and without face masks, finding that safe distances increase to 4 m during sneezes. Furthermore, Busco *et al.* (2020) coupled experimental and numerical methods to include the effects of head motion and pressure effects during sneezing. They found that sneezing under these realistic conditions increases the droplet cloud's size two to four times compared to coughing.

Several other numerical studies have investigated the possibility of droplet contamination in commonly encountered environments. One study investigated aerosol transport in classrooms with varying droplet sizes, source locations, and barriers; its authors found that large droplets predominantly deposit on surfaces, whereas a significant quantity of droplets smaller than 15 *μ*m exit through the ventilation system after 15 min (Abuhegazy *et al.*, 2020). Two more studies investigated the possibility of droplet-based virus transmission in restrooms. Li *et al.* (2020c) simulated the toilet flushing process and found that 40%–60% of virus particles can reach above the toilet bowl. Wang *et al.* (2020) found that urinals can promote droplet transmission during flushing. Both papers' findings support the necessity of wearing masks and improving ventilation in restroom facilities. Researchers have also focused on the effects of ventilation in these common indoor environments. Dbouk and Drikakis (2021) investigated how ventilation, and the placement of an air purifier, affect aerosol dispersion in elevators. Zhang *et al.* (2021) simulated aerosol transport in urban buses under different ventilation rates, with and without fresh air ventilation, and the impact of mask-wearing in this environment. Nazari *et al.* (2021) studied the role of ventilation in underground car parking structures, which can produce hazardous areas with respect to aerosol inhalation when walking through the car park.

Recent virologic and modeling results support the transmission possibility of COVID-19 disease from pre-symptomatic (symptom onset has not developed yet) or asymptomatic (symptoms of COVID-19 never develop) individuals (Li *et al.*, 2020a; Bai and Yao, 2020) to healthy humans. These findings reinforce the value of community precautions and regulations to slow the transmission of COVID-19 in environments where humans are in close contact with each other. Centers for Disease Control (CDC) suggests crucial steps, including physical distancing, face-covering, and universal masking in healthcare facilities to prevent transmission of COVID-19 disease from asymptomatic and pre-symptomatic individuals (Lasry *et al.*, 2020; Klompas *et al.*, 2020b). However, this reveals the vulnerability of healthcare facilities, especially when considering certain medical procedures where a face-covering is not practical, such as in dentistry, which might result in aerosol exhalation or generation in these environments.

Dental clinics are among the healthcare facilities that are particularly vulnerable to the COVID-19 disease. Dental care devices inevitably carry the risk of COVID-19 infection during their routine operating procedures through exposure to saliva, blood, and exhaled liquids from the patient's mouth. Additionally, the tools routinely utilized for dental procedures, such as ultrasonic scalers and drills, may generate fine droplets or aerosols. The pathogenic microorganisms, including the SARS-CoV-2 virus, can be transmitted in dental clinics by inhalation of airborne droplets or aerosols, direct contact with blood, oral liquid, or patient's nasal or oral mucosa. Also, indirect contact with contaminated instruments can cause infection. For infection control, specific guidelines are established to identify symptomatic patients and prevent servicing them (Peng *et al.*, 2020). The presence of asymptomatic and pre-symptomatic patients in dental clinics requires extra prevention steps to disinfect the instruments frequently and monitor the trace of airborne aerosols to prevent inhalation by patients and medical staff. Another compounding factor is the sustained proximity of practitioners to patients during dental procedures, which increases the risk of infection (Klompas *et al.*, 2020a). Although increased ventilation may reduce risks associated with aerosols (Somsen *et al.*, 2020), it alone is unlikely to mitigate all risks (Klompas *et al.*, 2020a).

The use of ultrasonic dental scalers is of particular interest in this study due to their wide employment in clinical dentistry settings. Ultrasonic scaling is useful for preventing periodontal diseases due to the effective removal of calculus and plaque from the tooth surface. However, prior studies have identified several risks associated with ultrasonic scaling (Trenter and Walmsley, 2003). The primary risk addressed in this study is the generation of fine aerosols during the scaling procedure, which may pose health risks (Suppipat, 1974). A study by Larato *et al.* (1967) found that counts of airborne bacteria increased 30-fold in clinics during ultrasonic scaling procedures. Similarly, the study of Legnani *et al.* (1994) found that the mean values of airborne microbial load increased threefold during working hours when using an ultrasonic scaler in a university clinic setting. Additionally, airborne microbial load remained 1.5–2 times greater than the control experiment after the conclusion of the scaling procedures at the end of the day. The findings suggested that patients treated toward the end of the day would be exposed to more microbiological contamination than those earlier in the day (Legnani *et al.*, 1994).

The goal of the current study is to assess the contamination risk of the SARS-CoV-2 virus in large, enclosed spaces. The study considers a large dental clinic at the University of Illinois at Chicago (UIC) when it is occupied by patients under realistic conditions. This goal is achieved by conducting computational fluid dynamics (CFD) simulations of the ventilated air flow inside the dental clinic and then tracking the trajectories of droplets and aerosols generated by an ultrasonic scaling device. The numerical results predict the patterns of airflow and droplets/aerosols movement inside the dental clinic and identify the locations with a high risk of contamination. This study can be used to provide guidelines for safer operations of healthcare facilities during the present and future pandemics for both medical staff and patients.

## II. THE DENTAL CLINIC AT UIC

**FIG. 1.**
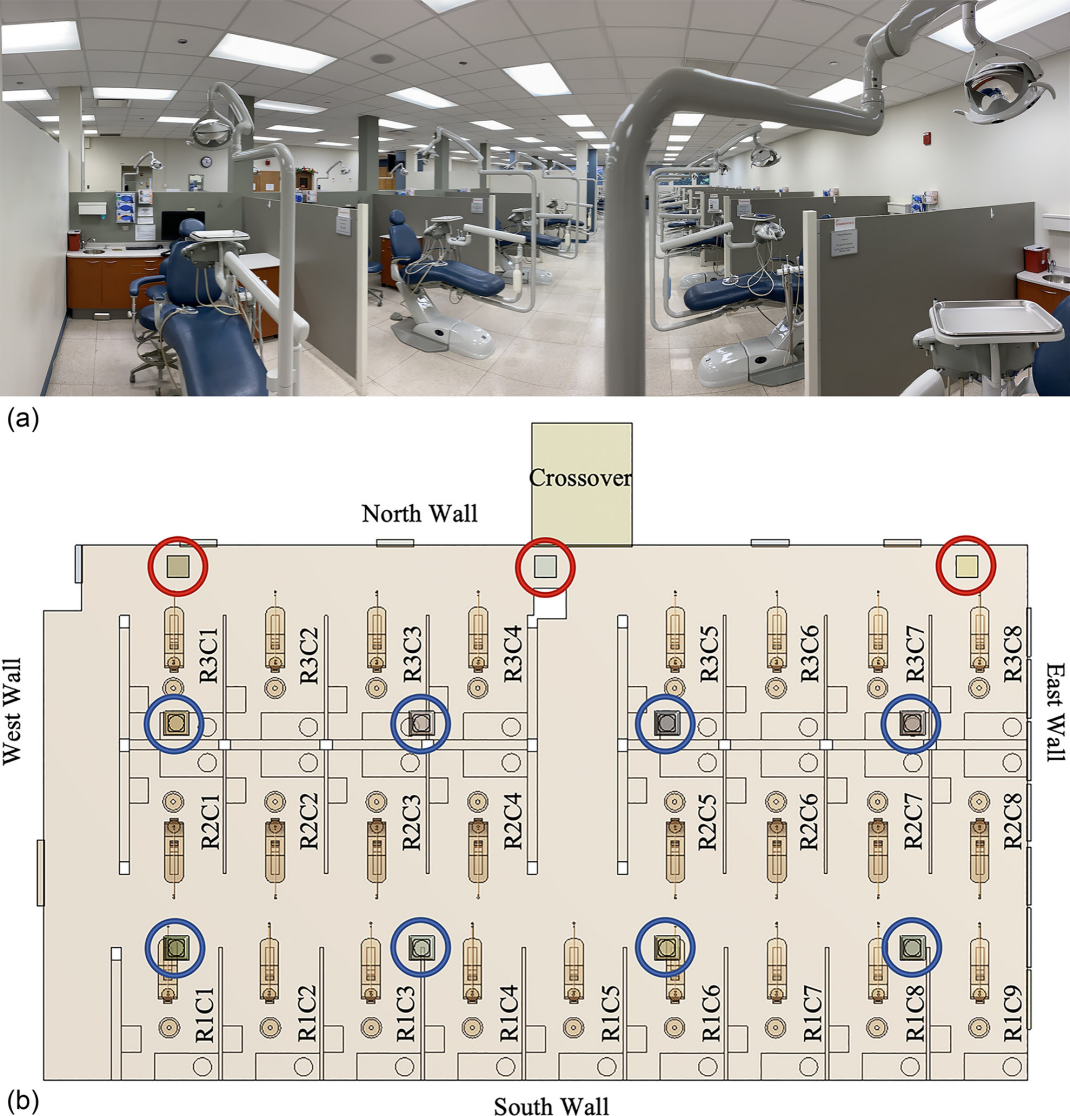
(a) Dentistry clinic and (b) layout of the dentistry room. Red and blue circles locate the ventilation outlets and inlets, respectively. Labels within the patient cubicles denote the cubicle number.

The simulation domain of this study is one of the dental clinics in the College of Dentistry (COD) at UIC. The particular clinic is selected because it is representative of five identically sized and equipped student clinics in the COD. The simulation results could therefore provide insights beneficial to the remaining four clinics. This clinic has a length × width × height = 24.1 × 13.1 × 3.0 m^3^ (corresponding to 79.2 × 43.0 × 9.8 ft^3^) operating environment. The floor and the suspended ceiling are made of 0.6 × 0.6 m^2^ porcelain tile and 0.6 × 0.6 m^2^ fiber tile, respectively [Fig. 1(a)]. The east wall [Fig. 1(b)] is exposed to the outside of the dentistry building and mounts seven large windows. The wall is composed of plaster, plastic foam insulation, concrete block, air gap, and precast concrete materials while the windows are made of tinted glass. The north, south, and west walls [Fig. 1(b)] toward the interior of the dentistry building are made of plaster panels.

**FIG. 2.**
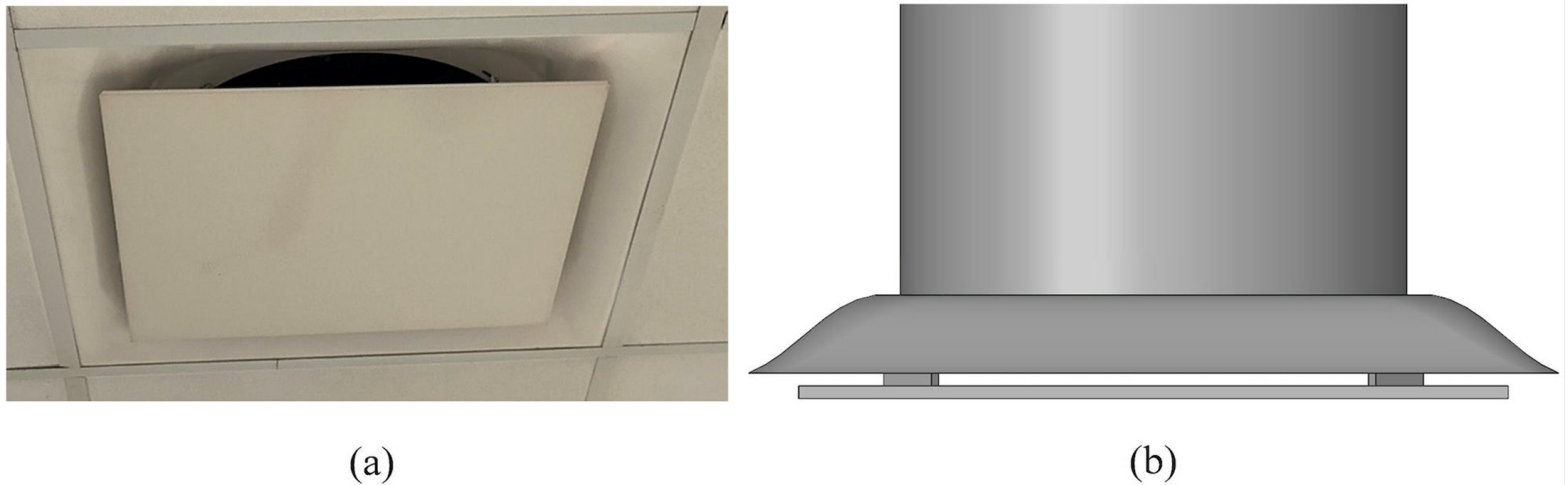
(a) Inlet vent mounted on the ceiling of the clinic and (b) CAD model of inlet vent.

The clinic's ventilation system is composed of eight inlet vents and three outlet vents [Fig. 1(b)]. Each inlet vent is designed as a diffuser (Fig. 2). Different inlet vents supply different inflow rates and temperatures of air. This clinic has a total area of 316 m^2^ divided into 25 dental cubicles by partitions [polyvinyl chloride (PVC) panels], each cubicle with 7.4 m^2^. Every cubicle is equipped with a dental unit, a dental assistant stool, and two cabinets located next to, and behind, the dental provider stool [Fig. 1(a)]. The dental unit is the primary unit of dental surgery equipment, which consists of a dental chair, a lamp, and a spittoon. Every dental unit has at least one high-speed handpiece, one low-speed handpiece, high and low volume evacuation system, and an air-water syringe. The unit is furnished with water through a system of tubes which are constituted with dental unit water lines.

**FIG. 3.**
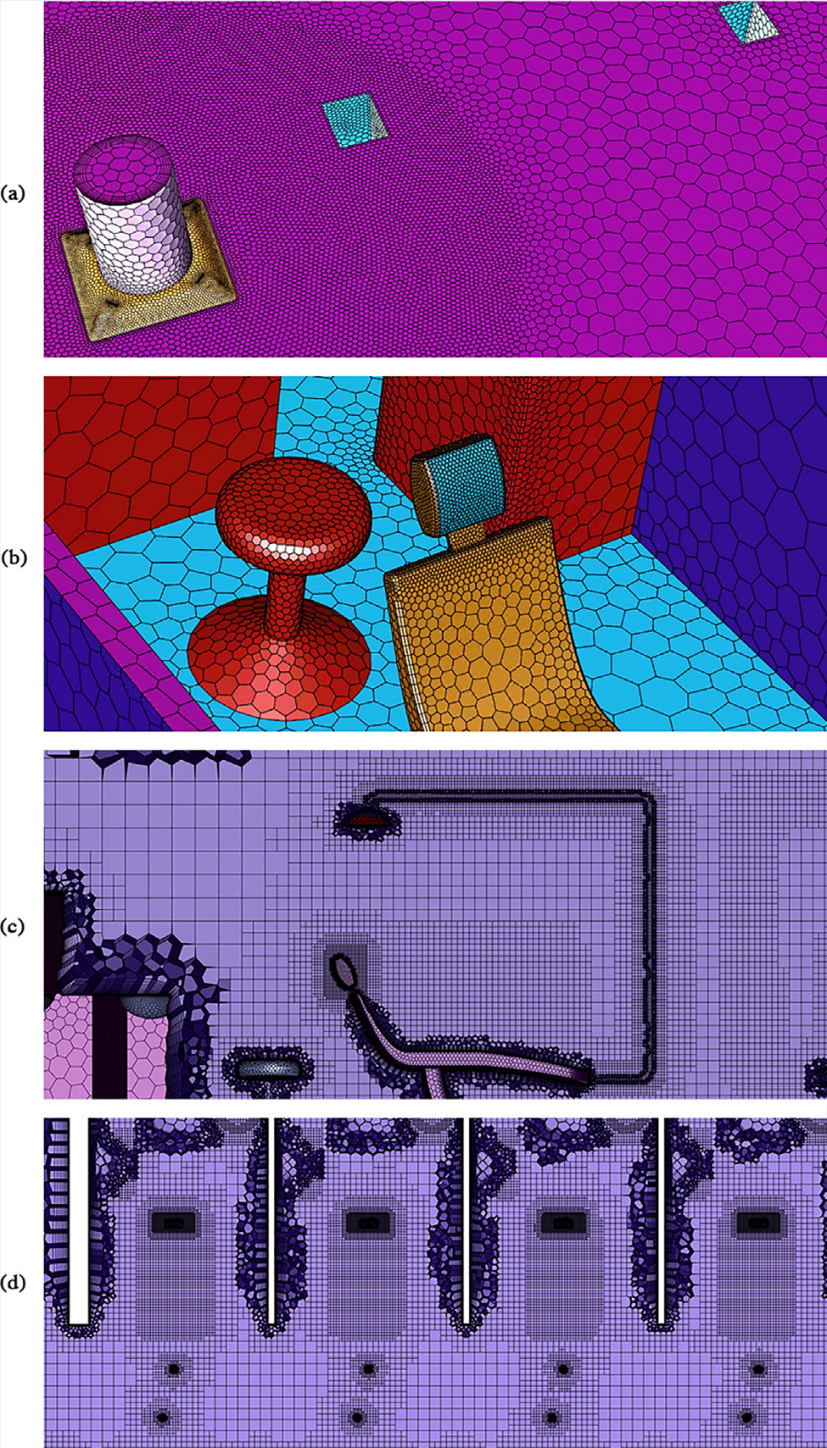
(a) Details of the numerical mesh near high-velocity inlets, (b) curvature refinement for dental unit, (c) side view of mesh refinement near the dental unit, and (d) top view of mesh refinement in the immediate vicinity of the patient chair.

The dental clinic was modeled in Solidworks based on blueprints and physical measurements of the actual dental clinic. Then, ANSYS Fluent Meshing (ANSYS, 2020) was used to generate the unstructured mesh for the computational domain. Due to the complex geometries of the inlet diffusers and the dental units, as well as the sharp velocity gradient near the inlets, we adopted advanced Fluent Meshing features (ANSYS, 2020) such as local sizing, body of influence, curvature refinement, and inflation layers (Fig. 3). We also simplified the dental unit with the consideration that it dramatically improves the mesh quality and keeps the necessary features of the dental unit. Consequently, high-quality meshes were built, which are characterized by low cell skewness and high orthogonality quality. The number of generated cells in the computational domain was significantly reduced, which largely lowered the computational cost.

To validate the mesh, we conducted a grid independence study, without droplets, over coarse meshing to dense meshing. The grid independence study showed that the fluid phase solution converged when the number of cells in the computational domain exceeded 9 × 10^6^. Additional cells were added in the computational domain to improve the quality and stability of the dispersed phase. We found that areas near the patient chair experienced large droplet source terms, due to droplet evaporation and settling, in the immediate vicinity of the droplet injection locations. Subsequent simulations were performed, while increasing the mesh resolution near the patient chairs, until a high-quality solution was achieved. The refined mesh near the injection source and patient chair are shown in Figs. 3(c)–3(d). The final mesh contains 20 545 103 cells. We also validated the numerical solution against our experimental measurements in the dental clinic. A detailed discussion of the validation study is given in Sec. IV C.

## III. SIMULATION APPROACH

The simulations utilize the ANSYS Fluent 2020R1 commercial software (ANSYS, 2020). For brevity, the governing equations employed for the simulations are excluded in this paper. However, the reader may reference the ANSYS Fluent documentation (Ansys Academic Research Mechanical and CFD, 2020d) for detailed descriptions of the models and their governing equations. Sections III A–III D describe the models used for the simulations, as well as the justification for their selection.

### A. Fluid flow

Determining droplets' behavior within the dental clinic requires simulating the continuous phase (airflow) and a discrete phase (droplets) dynamics (Feng *et al.*, 2020; Zhang *et al.*, 2019; Guan *et al.*, 2014; and Mashayek and Pandya, 2003). The airflow movement inside the room is governed by the conservation of total mass, water vapor mass, momentum, and energy, cast into the incompressible Navier–Stokes (NS) equations (Zhang *et al.*, 2019; Ye *et al.*, 2016). The indoor air is modeled as an ideal gas mixture of air and water vapor to account for the effect of humidity inside the room. Prior research demonstrated the importance of humidity on the evaporation of virus-laden droplets (Dbouk and Drikakis, 2020a; 2020c) and the viability of coronavirus virions inside the droplets (Bhardwaj and Agrawal, 2020). The humid air density varies with temperature to include the buoyancy force induced by temperature gradients. The incompressible Navier–Stokes equations are Reynolds-averaged in time to remove the need to calculate minute fluid movements (small scale eddies) (Pope, 2001). The Reynolds averaged Navier–Stokes (RANS) equations are completed by modeling the Reynolds stress and scalar mass flux terms consisting of velocity, temperature, and species fluctuations (Wilcox, 1993).

The RANS equations are solved employing ANSYS Fluent 2020R1 (ANSYS, 2020) commercial package. The common realizable k–ε model (Shih *et al.*, 1995) is selected for turbulence modeling since it provides an improvement in predicting both round and planar jet spread rates (Ansys Academic Research Mechanical and CFD, 2020f). It also demonstrates superior performance in capturing the effect of adverse pressure gradients on the boundary layer, separation, and reattachment regions (Ansys Academic Research Mechanical and CFD, 2020f). Note that the air velocity suddenly varies from the free stream speed to zero on the wall surface. Wall modeling can be beneficial in terms of computational cost since it decreases the number of computational cells required for resolving the high gradient zone near the walls. Therefore, we employ the Enhanced Wall Treatment (Jongen and Deville, 1998 and Kader, 1981) model to account for near-wall effects. The Enhanced Wall Treatment model ensures the validity of wall shear stresses due to the velocity and temperature gradients.

Six partial differential equations governing conservations of mass, momentum, turbulent kinetic energy (TKE) (k), turbulent dissipation rate (ε), water vapor species transport, and temperature are discretized in spatial coordinates to acquire the steady-state solution. The pressure equation is discretized using the pressure staggering option (PRESTO) scheme (Ansys Academic Research Mechanical and CFD, 2020a), whereas the other five equations employ the second-order upwind method (Barth and Jespersen, 1989). The pressure–velocity coupling occurs using the Coupled scheme (Ansys Academic Research Mechanical and CFD, 2020b), and the solution iteration is performed by the Pseudo Transient under-relaxation method (Ansys Academic Research Mechanical and CFD, 2020e) to ensure the stability of the solution. The steady-state solution is determined by an iterative process, starting from an initial guess for the steady solution in the physical domain. The initial guess in this study is set based on the hybrid initialization, which is performed by solving a Laplace's equation to determine the velocity and pressure field.

### B. Droplet transport

The transport of droplets and aerosols generated by the ultrasonic scaler is determined by the use of the discrete phase model (DPM) in ANSYS Fluent 2020R1 (ANSYS, 2020). The DPM uses a hybrid Euler–Lagrangian framework, where the Navier–Stokes equations are solved for the fluid phase, and the dispersed phase is solved by tracking a large number of droplet streams through the mean velocity flow field (Ansys Academic Research Mechanical and CFD, 2020c). Several recent notable numerical studies of the transport, dispersion, and evaporation of virus-laden droplets employ the DPM approach in Fluent (Li *et al.*, 2020b; 2020c; Abuhegazy *et al.*, 2020; and Feng *et al.*, 2020). The numerical droplet streams carry the mass flow rate of a greater number of physical droplets, though the trajectories are based on the masses and diameters of individual droplets. The solution employs a two-way coupling, where the mass, momentum, and energy are exchanged with the fluid phase. This exchange of information between the two phases ensures that the fluid flow field is updated with droplet sources, such as the evaporation of water from droplets contributing to humidity in the room. Due to the low volume fraction of the droplets/aerosols, and low mass loading, we neglect droplet–droplet interactions.

The DPM approach includes several external forces in order to predict droplet trajectories in the clinic setting accurately. The drag force acting upon the droplets follows the spherical drag law, where the constant coefficients are given by Morsi and Alexander (1972). The Morsi and Alexander model is selected due to its applicability across a wide range of Reynolds numbers. Due to the size of the droplets, we consider the thermophoretic effect suggested by Talbot *et al.* (1980). The thermophoretic force causes small droplets suspended in a fluid to experience a force opposite to the direction of the temperature gradient. Although the effect of thermophoresis can be considered negligible on large droplets in small temperature gradients, the thermophoretic force can affect small droplets. In this research, the thermophoretic force is only applied to the small, aerosolized droplets and droplet nuclei remaining in the room after evaporation. The droplets also experience the Saffman's lift force due to shear. The lift force utilized in this research is a generalization of the expression provided by Saffman (1965) and carries the form described by Li and Ahmadi (1992). Although additional forces may be considered, they are not included in this work. For example, the Brownian force is excluded since it is intended for sub-micron particles in laminar simulations, and the Magnus lift force is excluded as it is intended for large rotating droplets in high Reynolds number simulations (Ansys Academic Research Mechanical and CFD, 2020c).

The dispersion of droplets due to turbulence is modeled via a discrete random walk model (DRW). In the DRW model (Gosman and Ioannides, 1983), the instantaneous velocity is decomposed into the mean ($\bar{u}$) and fluctuating ($u^{\prime }$) components as $u=\bar{u}+u^{\prime }$. The mean velocity from the fluid phase is known at the droplet location, and the fluctuating component is sampled by assuming that it obeys a Gaussian probability distribution such that $u^{\prime }=\zeta \sqrt{u^{\prime 2}}$, where ζ is a normally distributed random number. The local root mean square (RMS) of velocities can then be related back to *k* in the k–ε turbulence model (Ansys Academic Research Mechanical and CFD, 2020c).

### C. Droplet evaporation model

Evaporation occurs if the droplet temperature exceeds the vaporization temperature, but remains lower than the boiling temperature $\left(T_{\mathrm{vap}}\leq T_{p}\leq T_{bp}\right)$. The droplet evaporation continues until there is no remaining volatile mass. Miller *et al.* (1998) and Sazhin's (2006) convection/diffusion-controlled evaporation model is utilized. The model is applicable at high vaporization rates and accounts for the effect of convective flow from the droplet's surface. The change in droplet mass follows

$$\begin{array}{c} \frac{dm_{p}}{dt}=k_{c}A_{p}\rho \ln\left(1+B_{m}\right), \end{array}$$ (1)

where $m_{p}$, $A_{p}$, t, and ρ are the droplet mass and surface area, time, and gas density, respectively. The Spalding mass number is given by

$$\begin{array}{c} B_{m}=\frac{Y_{s}-Y_{\infty }}{1-Y_{s}}, \end{array}$$ (2)

where $Y_{s}$ and $Y_{\infty }$ are the vapor mass fractions at the droplet surface and in the bulk gas, respectively. The mass transfer coefficient, $k_{c}$, is calculated through the Sherwood number correlation as

$$\begin{array}{c} Sh_{AB}=\frac{k_{c}d_{p}}{D_{v,m}}=2.0+0.6Re_{d}^{1/2}Sc^{1/3}, \end{array}$$ (3)

where $d_{p}$ is the droplet diameter, $D_{v,m}$ is the diffusion coefficient of vapor in air, and Sc is the Schmidt number. The Reynolds number based on droplet diameter is defined as $Re_{d}=\frac{\rho d_{p}\left|u_{p}-u\right|}{\mu }$, where μ is the fluid viscosity. The droplet Reynolds number is based on the relative velocity between the droplet, $u_{p}$, and the fluid phase, u. For low evaporation rates, the convection/diffusion model of Eq. (1) is expected to give similar results to the diffusion-controlled model (Ansys Academic Research Mechanical and CFD, 2020g).

The DPM uses a simple heat balance equation that relates the convective and latent heat transfer between the droplet and the continuous phase to the sensible heat change of the droplet (Ansys Academic Research Mechanical and CFD, 2020c)

$$\begin{array}{c} m_{p}c_{p}\frac{dT_{p}}{dt}=hA_{p}\left(T_{\infty }-T_{p}\right)-\frac{dm_{p}}{dt}h_{fg}. \end{array}$$ (4)

Here, $c_{p}$, $T_{p}$, and $h_{fg}$ are the specific heat, temperature, and latent heat of the droplet, respectively. Also, $T_{\infty }$ is the continuous phase's local temperature. The heat transfer coefficient, h, is evaluated through (Sazhin, 2006)

$$\begin{array}{c} Nu=\frac{hd_{p}}{k_{\infty }}=\frac{\ln\left(1+B_{T}\right)}{B_{T}}\left(2.0+0.6Re_{d}^{1/2}{Pr}^{1/3}\right), \end{array}$$ (5)

where Nu, Pr, and $k_{\infty }$ are the Nussult number, Prandtl number, and thermal conductivity of the fluid phase, respectively. The Spalding heat transfer number is defined as

$$B_{T}=\frac{c_{p,v}\left(T_{\infty }-T_{p}\right)}{h_{fg}-\frac{{\dot{q}}_{p}}{{\dot{m}}_{p}}},$$

where $c_{p,v}$ is the specific heat of the droplet vapor; ${\dot{q}}_{p}$ is the heat transferred to the droplet, and ${\dot{m}}_{p}$ is the rate of droplet evaporation. The Spalding heat transfer number can be related to the Spalding mass number of Eq. (2), through

$$\begin{array}{c} B_{T}={\left(1+B_{M}\right)}^{\frac{1}{Le}\;\frac{Sh}{Nu}\;\frac{c_{p,v}}{c_{p,g}}}-1, \end{array}$$ (6)

where Le is the Lewis number and $c_{p,g}$ is the specific heat of the gas mixture.

### D. Coupling of the fluid and dispersed phases

The two-way coupling is achieved by alternatively solving the fluid and dispersed phases. Three interphase exchange terms are calculated at specified iterations until the solution converges (Ansys Academic Research Mechanical and CFD, 2020c; Ansys Academic Research Mechanical and CFD, 2020g). The first interchange term is due to momentum exchange between the fluid and dispersed phases

$$\begin{array}{c} F_{\mathrm{mom}}=\sum \left[\frac{18\mu C_{D}Re}{24\rho_{p}d_{p}^{2}}\left(u_{p}-u\right)+F_{\mathrm{other}}\right]{\dot{m}}_{p}\Delta t, \end{array}$$ (7)

where $C_{D}$ is the drag coefficient (Morsi and Alexander, 1972), ${\dot{m}}_{p}$ is the mass flow rate of droplets, and $F_{\mathrm{other}}$ are the forces acting on the droplet, described in Sec. III B. The momentum exchange term appears as a source term in the fluid phase momentum balance. The second interchange term accounts for heat transfer between the fluid and dispersed phase. The change in thermal energy of a droplet as it passes through a cell follows:

$$\begin{array}{c} Q=\frac{{\dot{m}}_{p,0}}{m_{p,0}}[\left(m_{p,in}-m_{p,\mathrm{out}}\right)\left(-H_{\mathrm{lat},\mathrm{ref}}\right)-m_{p,\mathrm{out}}\int_{T_{\mathrm{ref}}}^{T_{p,\mathrm{out}}}c_{p,p}dT+m_{p,in}\int_{T_{\mathrm{ref}}}^{T_{p,in}}c_{p,p}dT]. \end{array}$$ (8)

Here, the subscripts p, 0, in, out, and ref denote droplet values, initial values, cell entry values, cell exit values, and reference values, respectively. $H_{\mathrm{lat},\mathrm{ref}}$ is the latent heat at reference values. If the cell is visited by multiple droplets, then Eq. (8) is calculated as a cumulative process of all droplets crossing the cell. The third interchange term adds mass to the fluid phase due to droplet evaporation

$$\begin{array}{c} M=\frac{\Delta m_{p}}{m_{p,0}}{\dot{m}}_{p,0}, \end{array}$$ (9)

where $\Delta m_{p}$ is the change in droplet mass. The change in droplet mass is calculated from the time the droplet enters the cell to the time it leaves the cell. Equation (9) is calculated as a cumulative process if multiple droplets contribute mass to the cell. The mass exchange term appears as a source of mass in the fluid phase continuity equation and the vapor species transport equation.

## IV. SIMULATION OF THE DENTAL CLINIC

### A. Boundary conditions (BC)

**FIG. 4.**
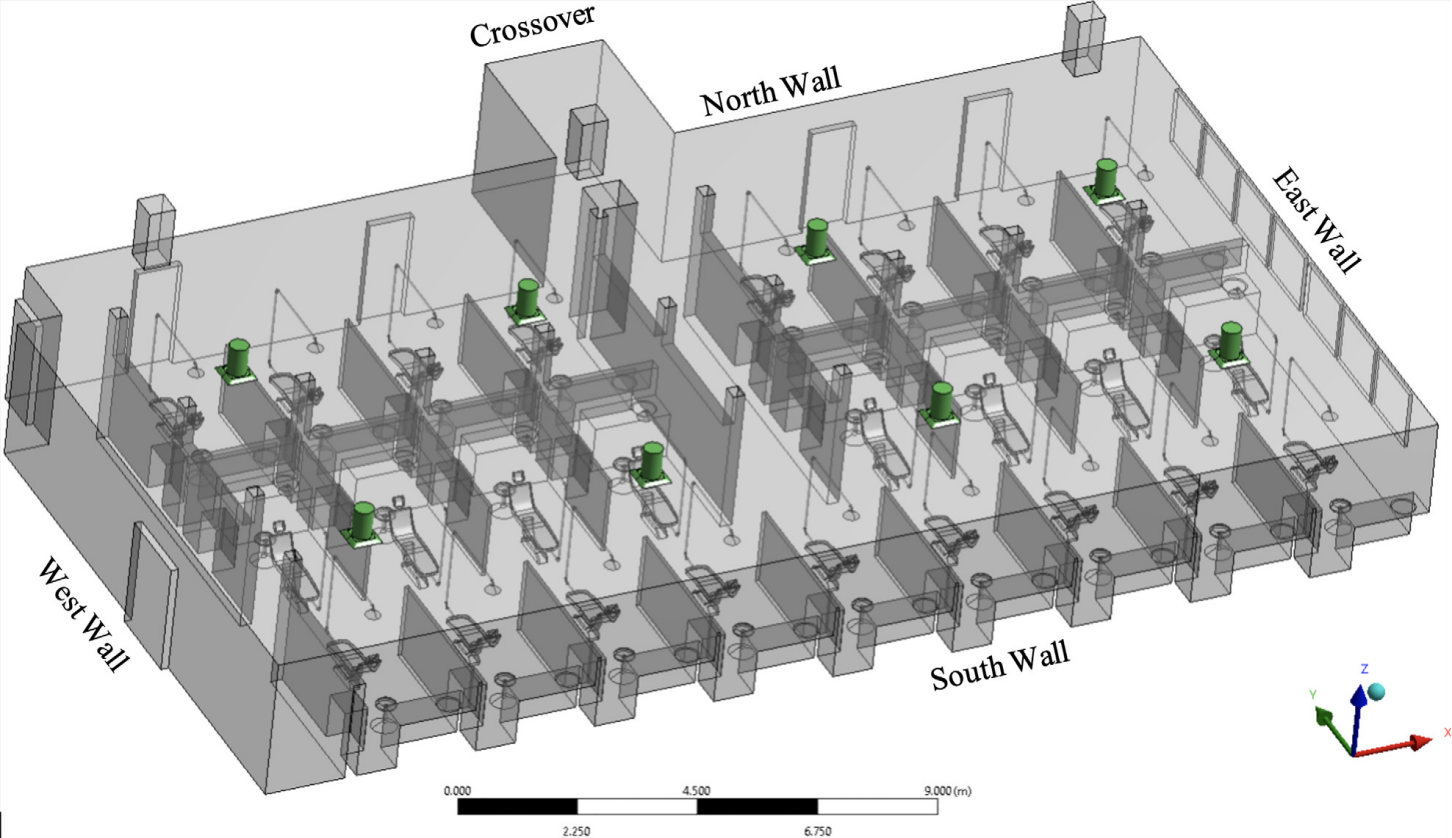
CAD model of the dentistry clinic. Conditioned air inlet vents are colored green.

A unique steady-state solution of the NS equations is determined based on the boundary conditions specified at the computational domain boundaries. The computational domain of the dentistry clinic is confined by the walls, equipment, inlet and outlet vents surfaces. The boundary conditions are specified by setting values for the temperature gradient on the wall surfaces, pressure on the outlet vents, and air mass flow rate entering the room from the inlet vents. For simplicity, we divide the boundaries into three categories: walls, inlet, and outlet surfaces. For each boundary, flow rate, thermal, and species conditions must be defined. A schematic of the dentistry clinic, along with all the relevant boundaries, is presented in Fig. 4.

#### 1. Wall boundary conditions

A wall boundary condition imposes the no-slip condition, meaning zero velocity. The physical boundaries defined as wall boundary conditions include exterior walls, interior walls, and interior objects' surfaces. The east and a portion of the south wall are exposed to the outside environment. These exterior walls consist of five construction material layers stacked from interior to exterior as plaster, plastic foam insulation, concrete block, air gap, and precast concrete layers. All the material layer properties, such as density (*ρ*), specific heat coefficient (*C_p_*), thermal conductivity (*λ*), and thicknesses (*t*), are given in Table I. The interior walls, including the west and north walls, are made of a single layer of 6-in. thick plaster. The ceiling is specified as a wall constructed of ceiling tiles, the windows are modeled as a glass wall, and the doors are assumed to be closed and constructed of a wooden layer (Table II for material properties). The floor is assumed to be a 15-in. thick layer of precast concrete. Other wall boundaries in the domain are considered as adiabatic walls with no thickness. The multi-layer exterior walls are modeled employing the shell conduction method (Ansys Academic Research Mechanical and CFD, 2020d) in Fluent.

**TABLE I.** Exterior walls material layers and properties.

Layer	$\rho \;(\mathrm{kg}/m^{3})$	$C_{p}\;(J/\mathrm{kg}\;K)$	$\lambda \;\left(W/m\;K\right)$	t (in.)
Plaster	849	900	0.892 4	¾
Plastic foam insulation	300	1300	0.030 9	1
Concrete block	2400	1000	0.413 0	4
Air gap	1225	1000	0.133 3	1
Precast concrete	2300	1000	0.262 2	6

**TABLE II.** Other wall boundaries and materials.

Boundary	Layer	$\rho \;(\mathrm{kg}/m^{3})$	$C_{p}\;(J/\mathrm{kg}\;K)$	$\lambda \;\left(W/m\;K\right)$	t (in.)
Interior Walls	Plaster	849	900	0.892 4	6
Windows	Glass	2500	840	0.094 1	1
Doors	Wood	740	1760	0.080 0	2

The thermal condition for the exterior walls, windows, interior walls, floor, and the ceiling is defined as heat convection to the exterior environment. The heat flux in these boundaries is set equal to the convection heat transfer rate calculated as

$${\dot{q}}_{|\partial \Omega }=h\left(T_{\infty }-T\right),$$ (10)

where ${\dot{q}}_{\left|\partial \Omega \right.}$ is the heat flux at the boundary of the physical domain (Ω) denoted as ∂Ω, h is the convection heat transfer coefficient, and $T_{\infty }$ is the exterior air temperature in Kelvin (Table III). The convection heat transfer coefficients were determined iteratively, to satisfy the boundary conditions on the surfaces, and were verified through comparisons of the simulation results with the measurements in the dental clinic. The partitions, dental chairs, lamps, and furniture surfaces are all defined as adiabatic wall BCs. The species boundary conditions for water vapor on all the walls is zero diffusive flux.

**TABLE III.** Convection heat transfer boundary conditions.

Boundary	$h\;(w/m^{2}\;K)$	$T_{\infty }({}^{\circ}C)$
Exterior walls	32.0	35.0
Windows	32.0	35.0
Interior walls	1.0	22.2
Ceiling	1.0	23.3
Doors	1.0	22.2
Floor	4.0	23.3

#### 2. Inlet, outlet, and symmetry boundary conditions

The conditioned air is supplied to the room through eight inlet vents, as depicted in Fig. 4. The inlet vent geometry includes the diffuser, the plate underneath, and the four tabs retaining the plate to the diffuser surface (Fig. 2). The accurate geometry of the inlet vent correctly captures the velocity profile of the air stream blowing into the room. A portion of the inlet supply duct, which is physically located in the ceiling, is also included in the computational domain to define a mass flow inlet BC on the circular vent surfaces. The inclusion of the additional section of the supply ducts allows for the development of turbulence in the vent before entering the room. The mass flow rate ($\dot{m}$), temperature (*T*), and relative humidity (ϕ) of the supply air are determined experimentally and set as boundary conditions as shown in Table IV. The inlet vents are numbered from the window side of the room. The inlets' gauge pressure is zero, and the turbulence boundary condition is set by defining turbulent intensity and turbulent viscosity ratio. Turbulent intensity is described as the ratio of root mean square of the velocity fluctuations to the mean flow velocity. The turbulent intensity and the turbulent viscosity ratio are set to 5% and 10%, respectively, for all the inlet vents. The values for turbulent intensity and turbulent viscosity were selected based on the good agreement with experimental measurements of velocity and temperature near the outlets of the clinic's air inlet vents.

**TABLE IV.** Inlet boundary conditions.

Inlet vent	$\dot{m}\;\left(\mathrm{kg}/s\right)$	T (K)	$\phi \;\left(\%\right)$	$Y_{H_{2}O}\;({\mathrm{kg}}_{H_{2}O}/{\mathrm{kg}}_{\mathrm{mix}})$
South 1	0.203 2	287.15	81.3	8.16 × 10^–3^
South 2	0.242 6	287.09	82.0	8.20 × 10^–3^
South 3	0.208 7	292.71	65.6	9.38 × 10^–3^
South 4	0.195 9	292.76	64.6	9.26 × 10^–3^
North 1	0.261 1	286.98	83.0	8.24 × 10^–3^
North 2	0.260 4	287.09	81.0	8.10 × 10^–3^
North 3	0.196 9	292.59	64.3	9.12 × 10^–3^
North 4	0.190 0	292.59	64.6	9.17 × 10^–3^

The three outlet vents, as shown in Fig. 4, are extended to prevent the backflow due to fluid circulation at the outlet. The outlet surfaces are defined as pressure outlet boundary conditions with the gauge pressure of 0 Pa. The crossover surface to the other dentistry clinic (Fig. 1) is defined as a symmetry boundary condition due to a similar room on the opposite side of the crossover with the identical flow and thermal conditions.

### B. Droplet setup

#### 1. Droplet boundary conditions

The DPM approach requires the specification of droplet boundary conditions on all surfaces, inlets, and outlets. The droplet boundary conditions for all surfaces are specified as trapped (Ansys Academic Research Mechanical and CFD, 2020c). In the trap particle boundary condition, a droplet in contact with a surface is deposited on the surface. The volatile fraction of the droplet will evaporate, leaving only the nonvolatile fraction deposited on the surface, much like reality. The inlets and outlets are specified as escape boundary conditions. The escape boundary condition allows the droplet to leave the computational domain. Droplet mass exiting the domain is monitored as a convergence criterion. Once escaped and evaporated droplet mass is constant, we may assume the droplet trajectories are no longer changing within the domain, leading to a converged DPM solution.

#### 2. Droplet initial conditions

**FIG. 5.**
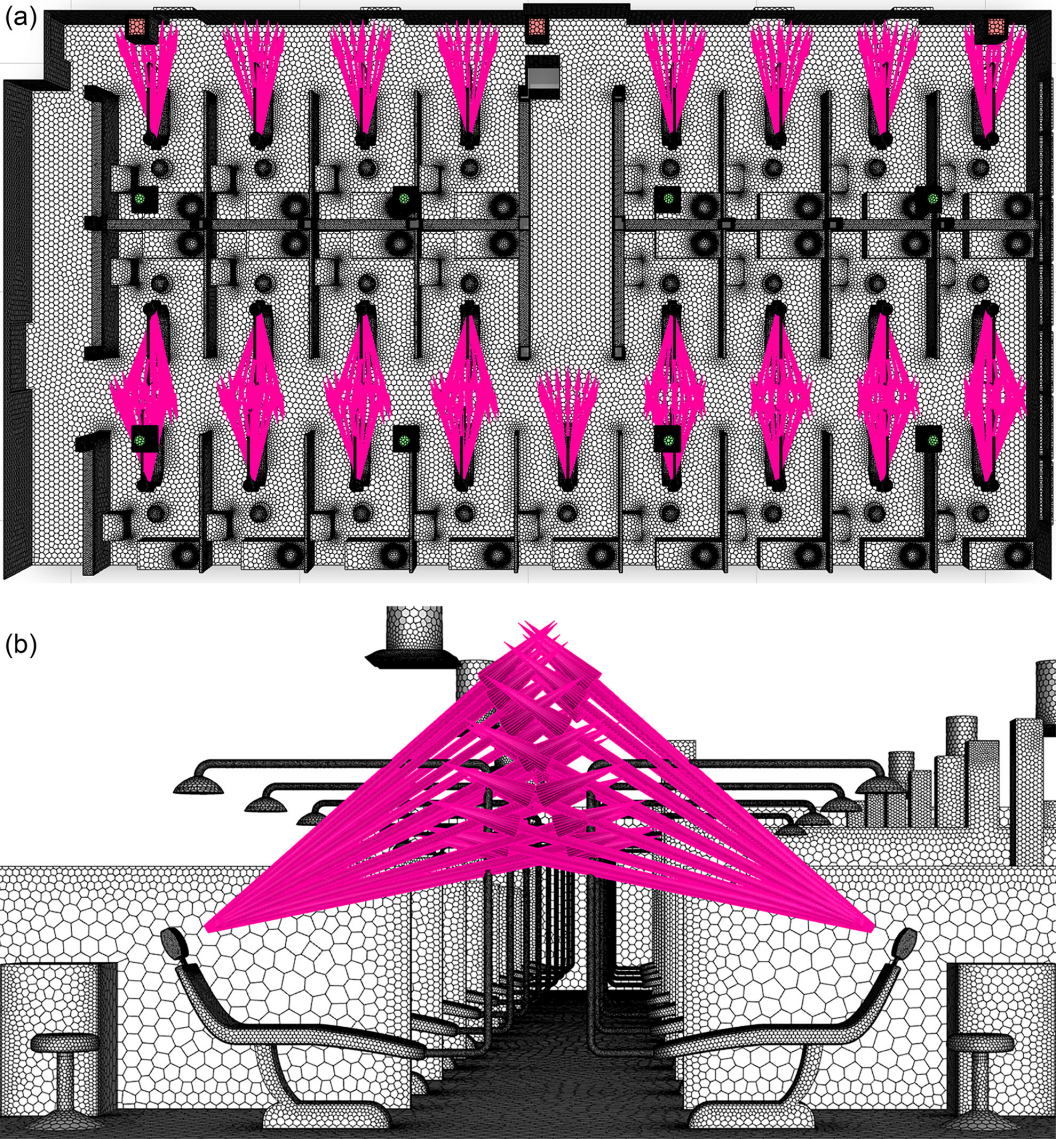
(a) Top view of droplet injection locations in the dentistry clinic and (b) side view of the injection away from the headrest location.

The injection locations for droplets correspond to the locations of the mouths of patients when seated in the dental chairs during an ultrasonic scaling procedure. In the full clinic setting, there are a total of 25 possible injection locations, corresponding to the maximum number of patients. Figure 5(a) depicts the injection locations within the clinic. The injection points are taken to be 1.2 m from the clinic's floor, the approximate height of the human mouth when positioned in the chair. Additionally, the distance from the front-center of the headrest to the injection is 0.20 m, as shown in Fig. 5(b). This distance corresponds to the mean distance of the glabella to the back of the head for the average human male adult (Young, 1993). Finally, the injection is modeled as a cone with a 15° half-angle, where the cone's central axis is normal to the headrest.

**FIG. 6.**
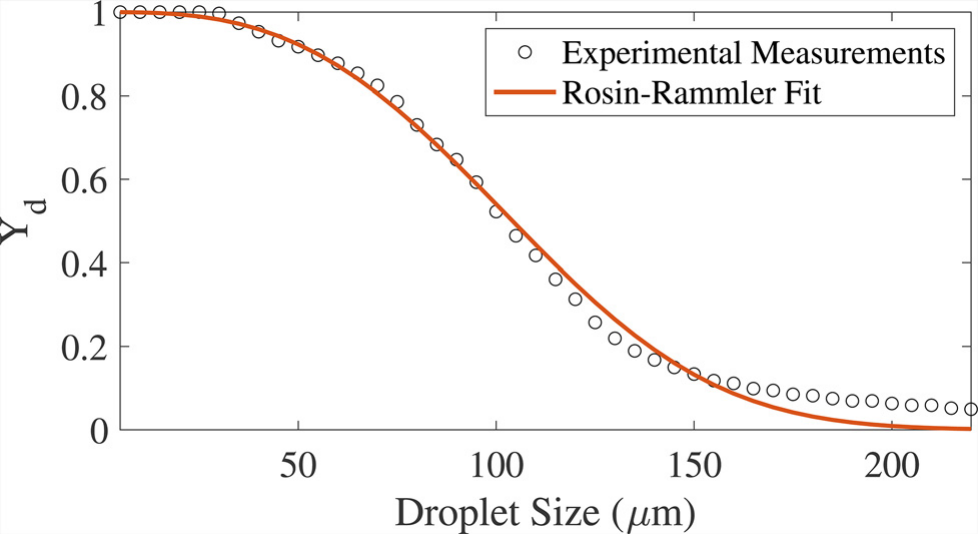
Experimental droplet size distribution of ejecta from patient's mouth compared to the Rosin–Rammler distribution used in DPM simulations.

The droplet size at the time of primary injection follows the distribution from the experimental measurements of Haffner *et al.* (2020) for a common commercially available ultrasonic dental scaling device, shown in Fig. 6. The experimental measurements are taken from a Cavitron ultrasonic scaling device at a flow rate of 16.2 ml/min, a typical setting used in dental practices. A Rosin–Rammler fit, which is commonly used to describe particle distributions, is applied to the experimental data (Brown and Wohletz, 1995 and Rosin and Rammler, 1933). The fit is shown to obey $Y_{d}=e^{{-\left(\frac{d}{\bar{d}}\right)}^{n}}$, where d is the bin's droplet diameter, $\bar{d}$ is the mean diameter, and n is the spread parameter. Applying the Rosin–Rammler fit to the data yields a minimum droplet size of 20 *μ*m, maximum droplet size of 220 *μ*m, a mean of 118 *μ*m, and a spread parameter of 2.93 as shown in Table V. The Rosin–Rammler fit produces a good approximation of the experimental data, although slightly under-predicting the frequency of larger droplets (Fig. 6). The droplet velocities are specified as the average velocity from the measurements of Haffner *et al.* (2020), producing a mean velocity of 0.68 m/s. The mass flow rate of droplet ejecta, shown in Table V, is the mass of droplets produced by the ultrasonic scaling device for 1 s. We note that the DPM injection properties and droplet size distribution depend on the settings of the Cavitron ultrasonic dental scaling device when in use. Haffner *et al.* (2020) also characterized the flow properties of the ultrasonic scaler at a higher setting of 30 ml/min and found differing droplet size distributions and velocities that are not considered in this study.

**TABLE V.** DPM Particle Conditions.

Condition	Value	Units
Mass flow rate	0.000 27	kg/s
Minimum drop size	20	*μ*m
Maximum drop size	220	*μ*m
Mean drop size	118	*μ*m
Spread parameter	2.932 9	
Temperature	310.15	K
Velocity	0.68	m/s
Volatile mass fraction	96.723 2	%

The droplet composition consists of volatile and nonvolatile mass. The volatile mass fraction consists of water, which evaporates, and a nonvolatile droplet nucleus that remains. Aerosols of droplet nuclei are considered important in studying the airborne transmission of viruses due to their small size, the concentration of virions, and their ability to remain airborne for extended periods of time (Vejerano and Marr, 2018). Several studies have focused on determining the rate of evaporation and the final diameter of human respiratory droplets (Vejerano and Marr, 2018; Duguid, 1946; and Liu *et al.*, 2017). For this study, we utilize the estimates provided by Liu *et al.* (2017), where the evaporated droplet nucleus diameter is approximately 32% of the initial droplet diameter for the relative humidity ranges considered in the simulations (Table IV). The volatile mass fraction may be calculated as a function of the initial and final droplet diameter, given in Table V. To the authors' best knowledge, more accurate estimates of the evaporated droplet nucleus size from an ultrasonic scaler are not available in the literature. Thus, we assume the remaining droplet nucleus will be of similar size to those expelled during coughing since the fluid from the scaling procedure will come into contact with the patient's saliva during the procedure.

### C. Validation of the dentistry clinic simulation

**FIG. 7.**
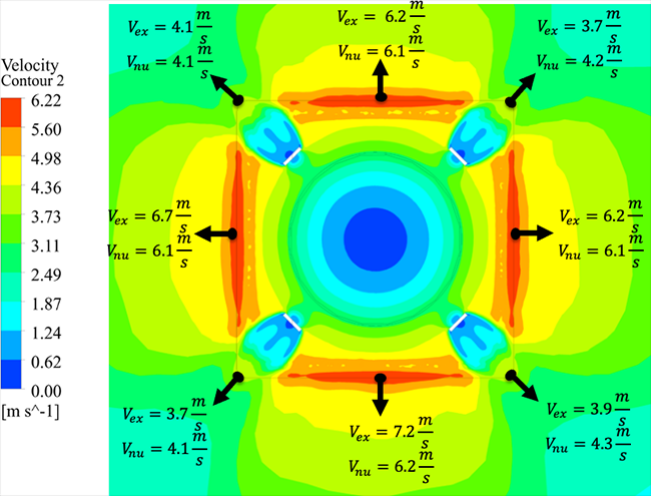
Comparison of the experimental measurements and numerical predictions for velocity exiting the vent in the clinic. Experimental measurements are $V_{ex}$ and numerical results are $V_{nu}$.

Prior to the simulation of aerosol-generating procedures in the dental clinic, we performed a validation study to ensure the numerical model corresponds to the real dentistry clinic conditions. Experimental and numerical measurements of velocity were taken at pre-determined locations surrounding the inlet vents (i.e., corners and sides at the location of the vent), as well as one foot from the vent outlet. The velocities were then compared between the numerical predictions and experimental results, as shown in Fig. 7. It was found that the inclusion of the tabs securing the vent bottom plate to the diffuser body was necessary to more accurately capture the low-velocity regions at the corners of the vents. Overall, the numerical velocities were within 10% of the experimental measurements across all vents in the clinic simulation using the boundary conditions prescribed in Table IV.

**FIG. 8.**
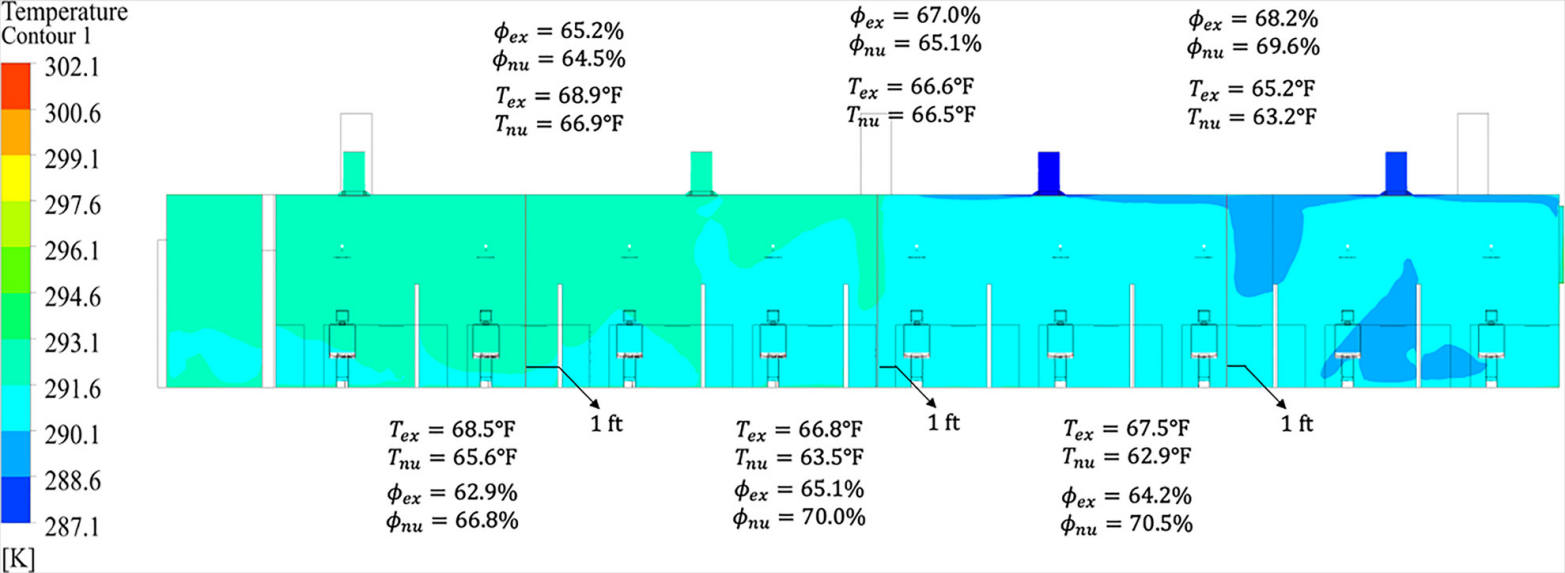
Comparison of experimental measurements and numerical predictions for temperature (T) and humidity (ϕ) at six locations in the clinic. Upper predictions are taken 1 in. below the drop ceiling and the bottom values are taken one foot from the floor.

Further validation consisted of comparing the temperature and humidity in the dentistry clinic. A total of six discrete measurements were taken in the clinic. The locations selected for measurement were between the inlet vents due to the mixing of air streams from different vents. At each intersection, the temperature and humidity were measured 1 in. from the ceiling, and one foot from the floor. The temperature and humidity from the experimental measurements were then compared to the numerical predictions as depicted in Fig. 8. The near-ceiling temperatures agree excellently, within 3%, between experimental measurement and numerical prediction, due to accurate modeling of the ceiling tiles and gap in the drop ceiling. Similarly, the temperature agreement near the floor was found to be within 7.3%, a slight increase in error, likely due to the unknown construction and thermal boundary conditions of the floors. The magnitude of humidity difference between experimental and numerical results was found to be less than 6.3%. Since relative humidity is dependent on temperature, a slight compounding of error is expected; however, the humidity is still well within the acceptable range for the droplet nonvolatile fraction assumption from Sec. IV B 2 to be considered valid.

## V. RESULTS AND DISCUSSION

Analyses of the air flow within the dental clinic room, as well as the associated droplet movement within the clinic, are presented in Secs. V A and V B. First, we discuss the solution of the Eulerian field, consisting of air and water vapor (humidity). We then use this analysis to understand the effect of the ventilation in the room on droplets generated due to dental procedures, such as ultrasonic scaling.

### A. Analysis of the flow in the dental clinic

**FIG. 9.**
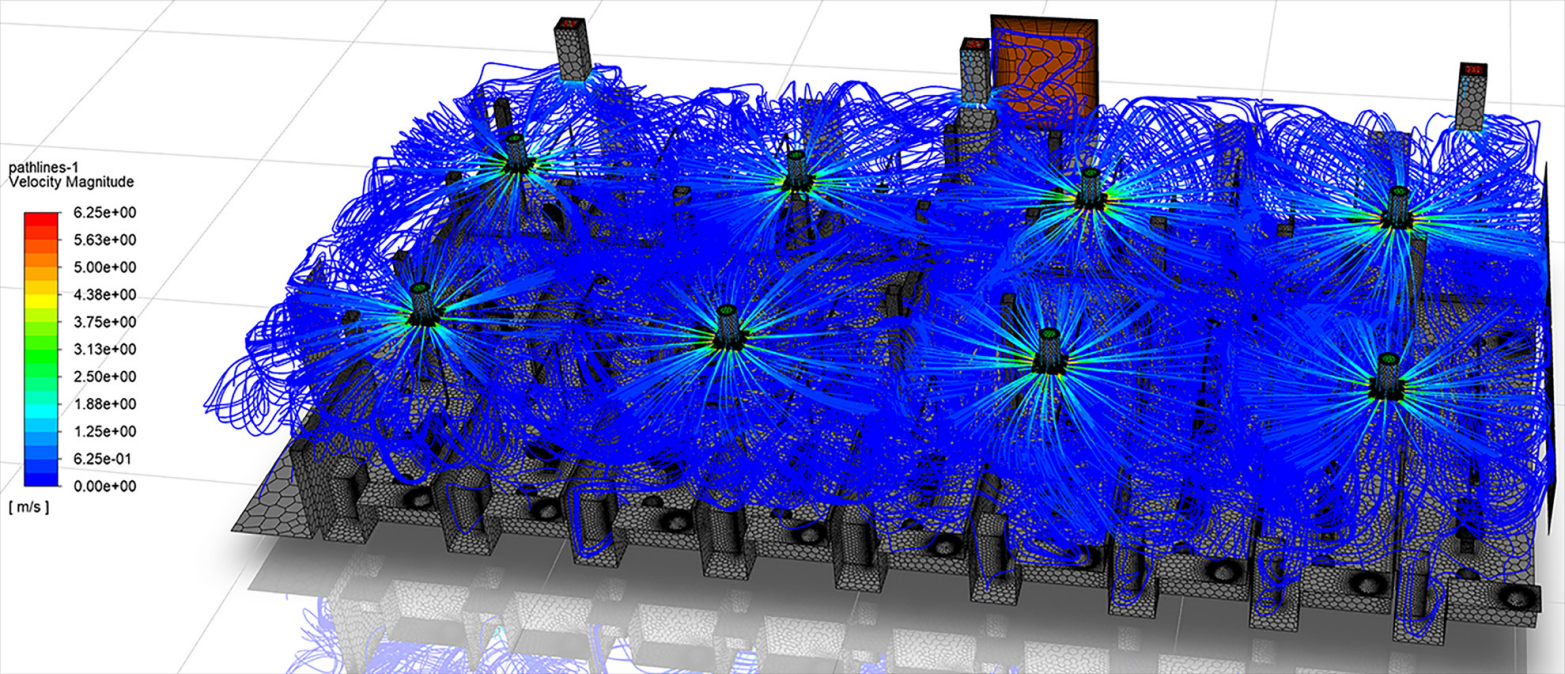
Streamlines colored by velocity magnitude in the dentistry clinic.

The dentistry clinic described in Sec. IV contains eight air vents that supply cool air through rectangular diffusers attached flush to the ceiling [Fig. 2(a)]. This style of ventilation produces attached jets, commonly known as wall jets. The wall jet forms when the air discharges close to the plane of the ceiling and the presence of the adjacent surface prevents entrainment of additional air, resulting in a pressure difference across the jet (Hagstrom *et al.*, 1999). The pressure difference curves the jet until it attaches to the ceiling, a phenomenon commonly referred to as “Coanda” effect (Hagstrom *et al.*, 1999; Saadeddin, 2016). This effect is observed in the velocity streamlines of the dentistry clinic, shown in Fig. 9. The cool air exits the vent at velocities up to 6.25 m/s, attaches to the ceiling, and travels along the ceiling surface. The jet then continues along the attached surface until separation. Jet separation from the ceiling may occur due to the downward buoyance force exceeding the upward Coanda force, the interaction of opposing jets, or the impingement of the jet with another surface (Hagstrom *et al.*, 1999).

**FIG. 10.**
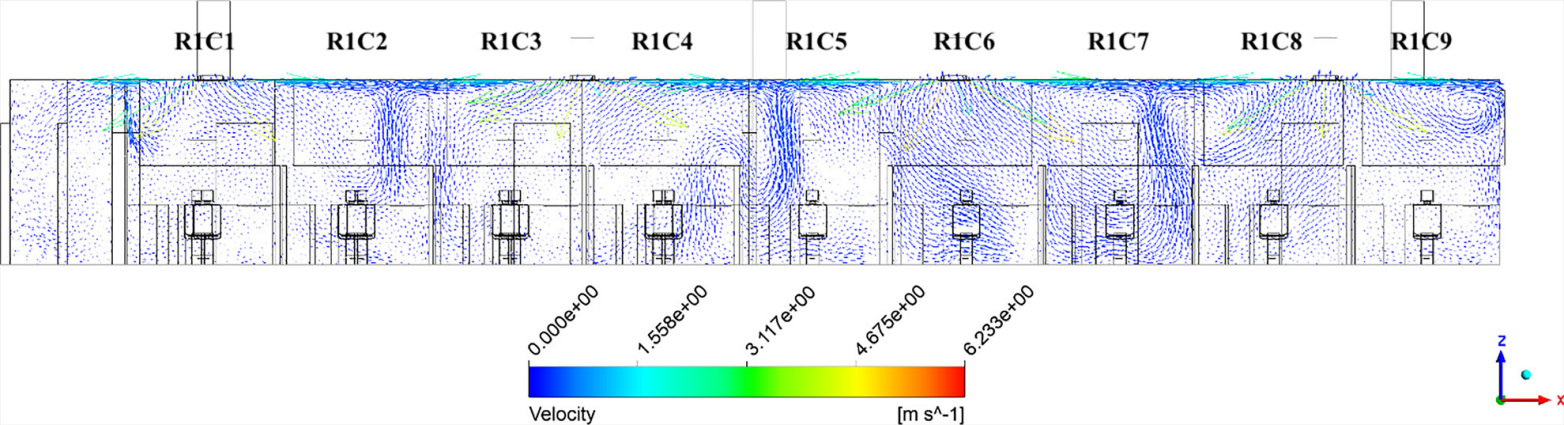
Velocity vectors along the vertical plane of the south vents in the clinic.

In this study, the dominant separation cause observed in the dentistry clinic is due to opposing parallel jets interacting, creating a downward traveling detached jet. This behavior may be observed in the velocity vectors of Fig. 10, where the vectors are plotted along a vertical plane intersecting the middle of the south inlet vents. In the figure, a strong downward velocity occurs at the midpoint between vents, creating detached jets. The three detached jets enter patient cubicles R1C2, R1C5, and R1C7, where they are observed to recirculate. The jets increasingly penetrate the cubicles from the west- to east-side of the room due to the higher vent mass flow rates on the east side of the room (Table IV). Two additional detachment locations occur due to impingement with a column and wall on the west- and east-sides of the clinic, respectively. The impingement of the jet with the wall on the east side of the clinic creates additional recirculation regions.

**FIG. 11.**
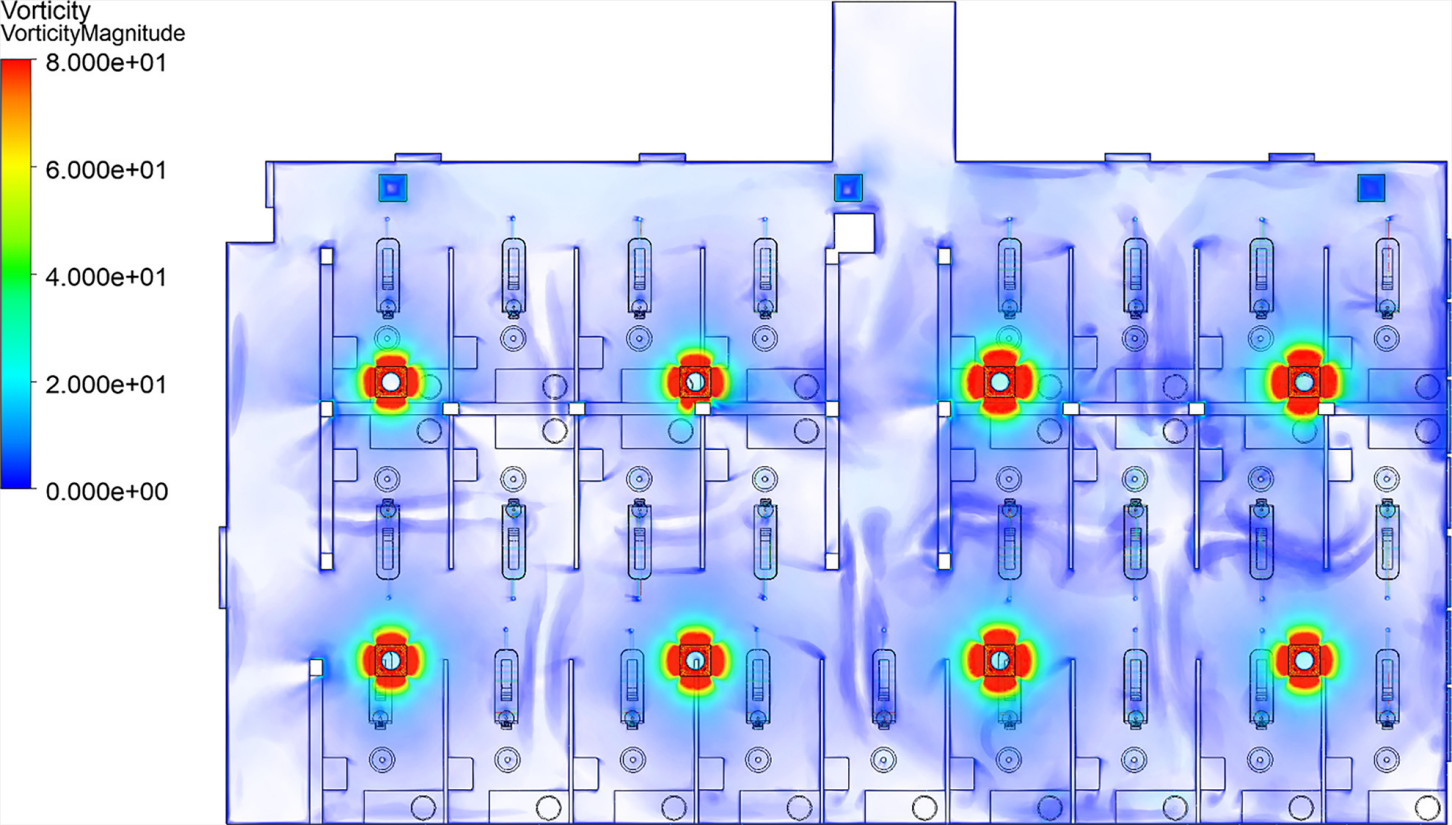
Vorticity magnitude in the dentistry clinic. Vorticity scale is limited to 80 [1/s].

**FIG. 12.**
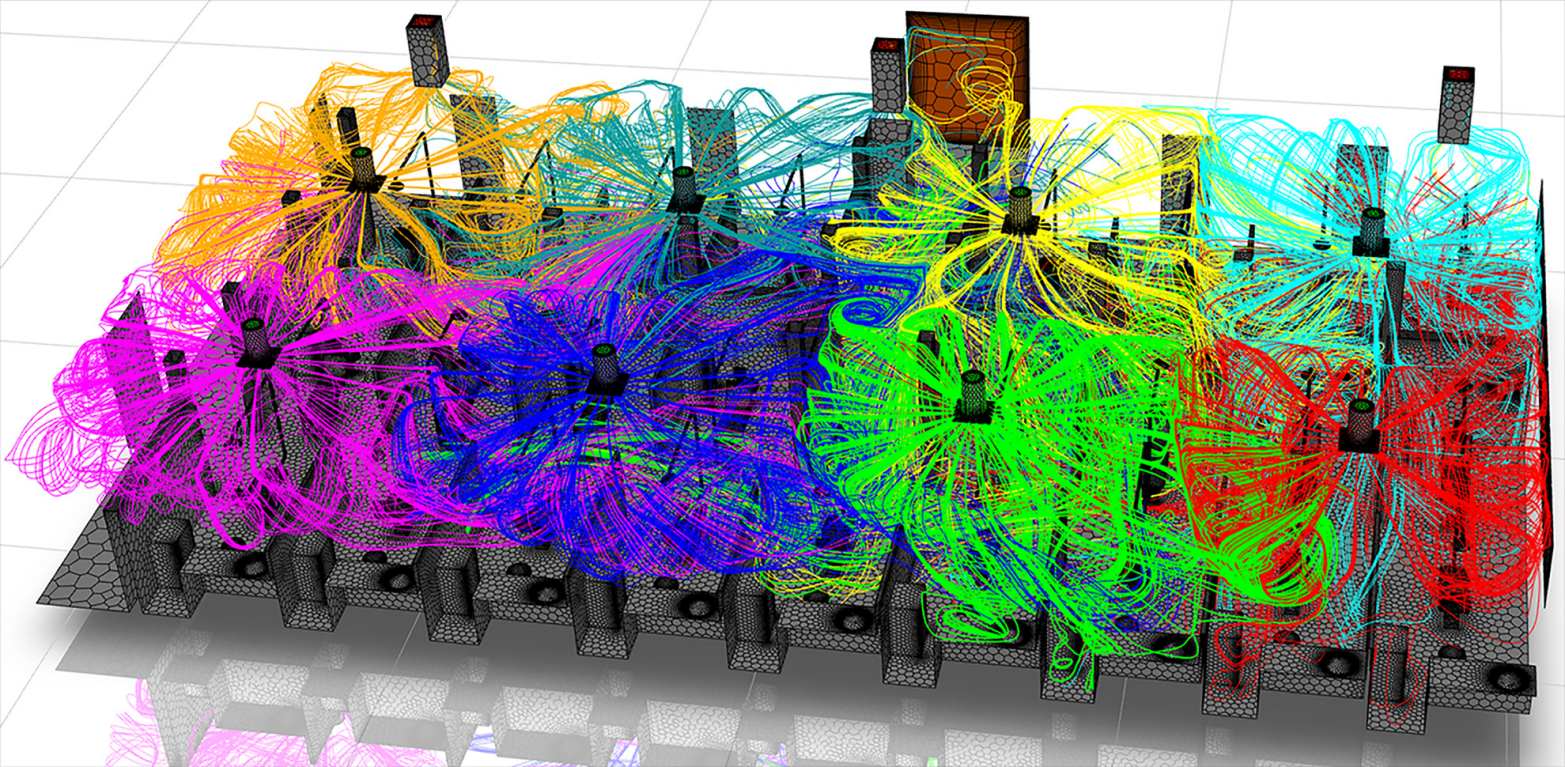
Streamlines colored by originating vent. Each color represents air jets entering the room from a specific vent.

To better understand the detachment regions in the clinic, we utilize the vorticity volume renderings of Fig. 11. Locations where the jet detaches from the ceiling are observed by low vorticity magnitude (transparent in Fig. 11). Specifically, locations where two jets interact and detach can be seen as areas where vorticity increases, then suddenly reduces to near-zero (transparent), such as the regions mid-way between opposing vents. In the absence of an opposing jet, the streamlines impinge on the dentistry clinic walls and then detach (Fig. 12). The detachment of these jets, either by impingement with a surface or interaction with another jet, creates eight zones within the room with return flows similar to that induced by a single jet in a smaller room. This behavior may be clearly observed in Fig. 12, where the streamlines are colored by the originating vent. Areas dominated by a single color of streamline in Fig. 12 represent a zone. Additionally, due to the exhaust vents being located on the north side of the room, the jets from the south-side of the room are observed to travel north toward the exhaust vents, causing additional mixing between the eight zones in the room.

**FIG. 13.**
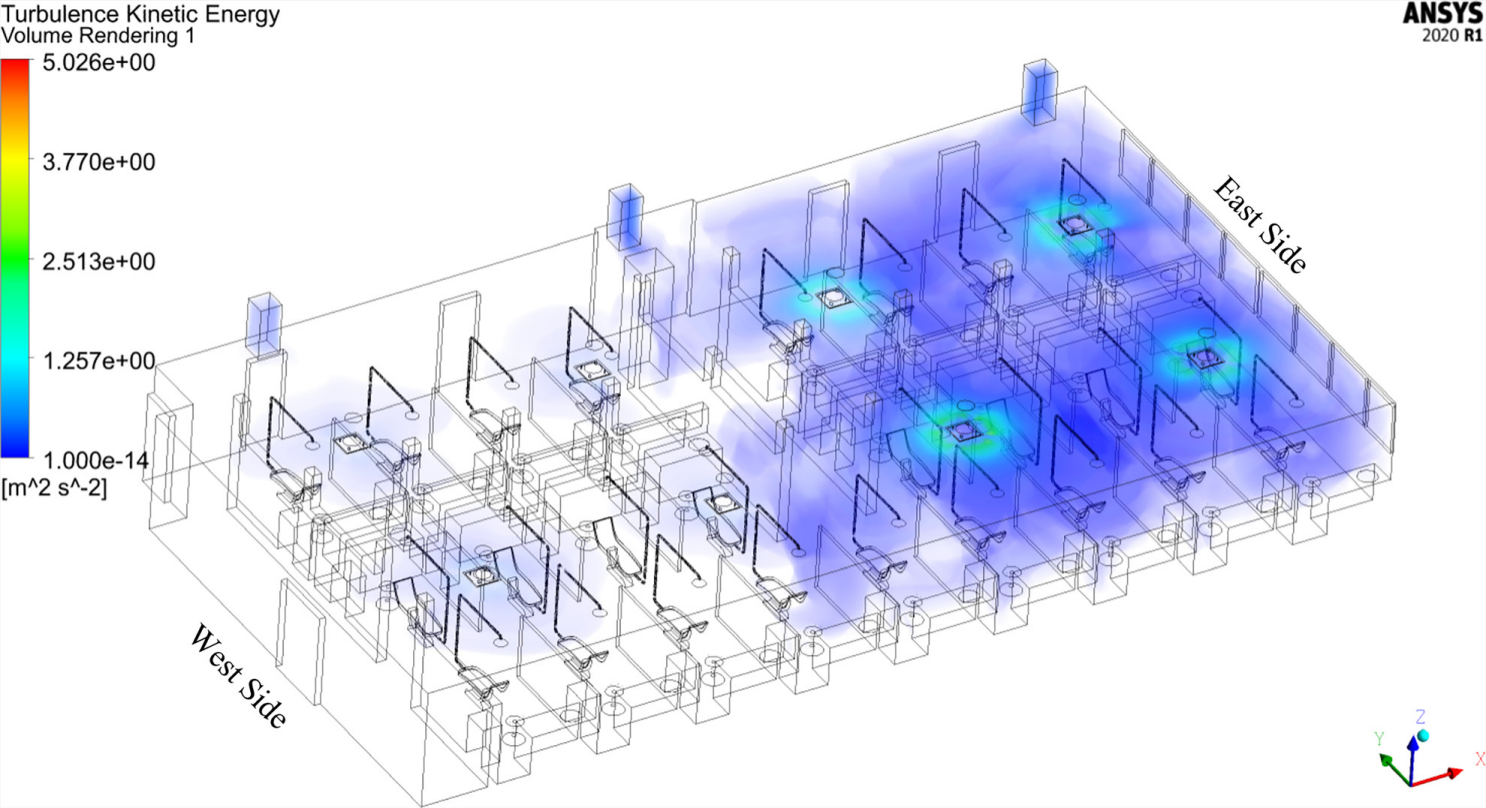
Turbulent kinetic energy in the dentistry clinic.

Furthermore, the results exhibit a non-uniform distribution of turbulence within the dental clinic. Figure 13 depicts the distribution of turbulent kinetic energy (TKE). From a physical perspective, a high TKE magnitude indicates the presence of strong RMS velocity fluctuations that can be produced by shear, friction, or buoyancy. We observe increased TKE in the east side of the room (window side). This increased TKE stems from the higher vent flow rates on the east side of the room (Table IV), which are likely due to an increased need of cooling due to sunlight. Similarly, a mild increase in vorticity magnitude appears in the east side of the room, shown in Fig. 11. The disparity in TKE and vorticity magnitude between the west- and east-sides also demonstrates an imbalance in the flow properties across the room, which influences droplet behavior, as discussed in Secs. V B 1–V B 4.

### B. Analysis of the droplet transport in the dental clinic

**FIG. 14.**
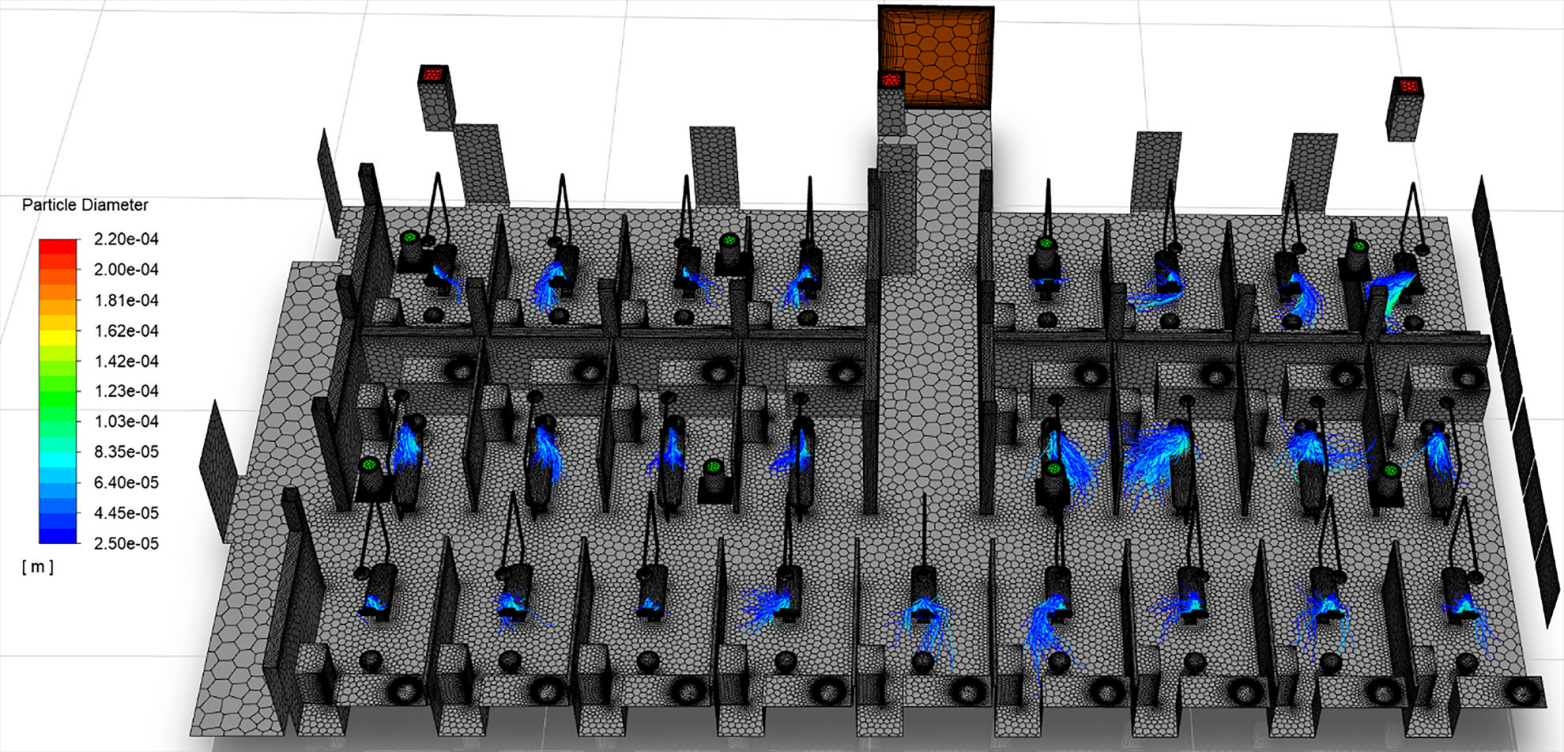
Transport of droplets over 25 *μ*m in diameter. Particle paths are colored by droplet diameter.

**FIG. 15.**
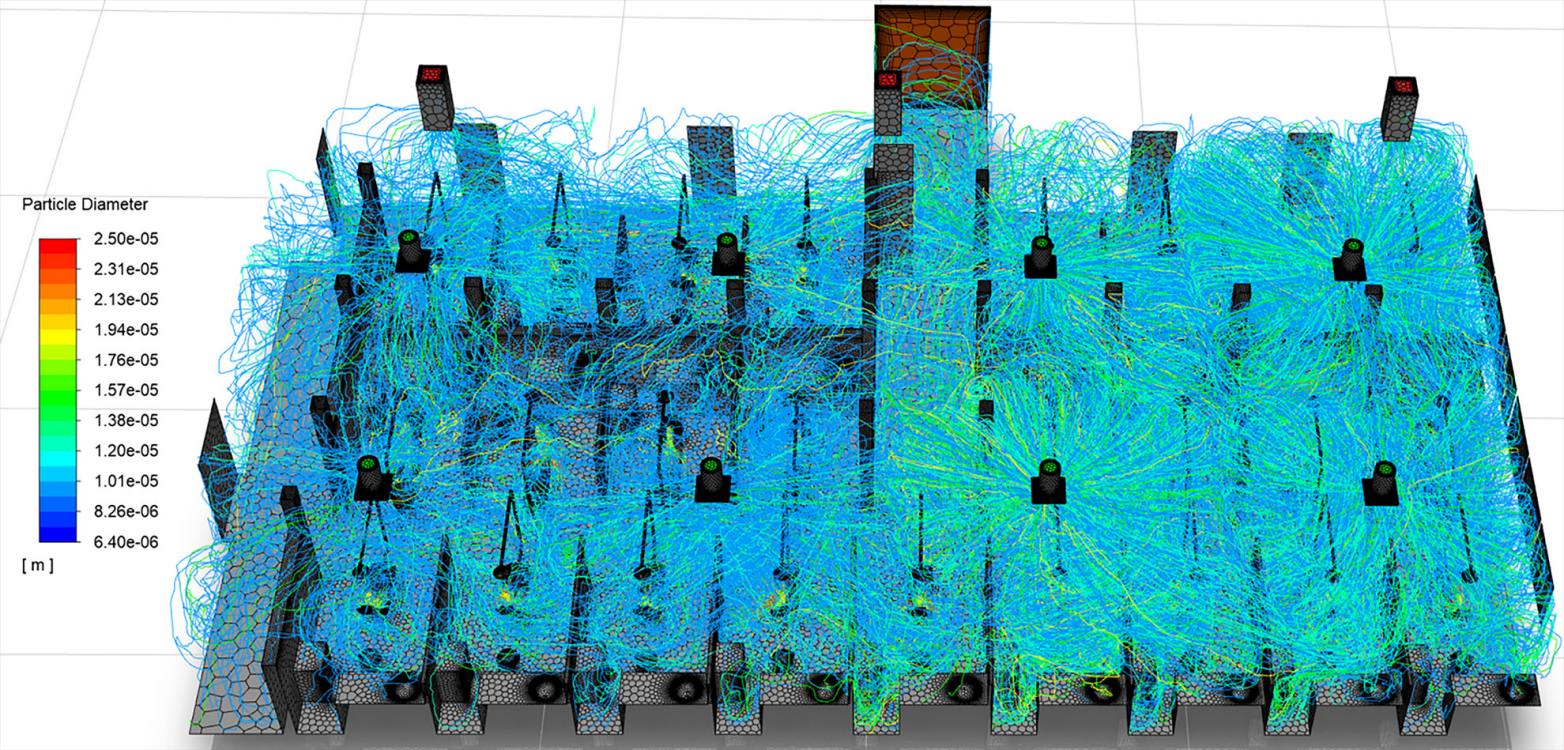
Transport of droplets under 25 *μ*m in diameter. Particle paths are colored by droplet diameter.

Visualizations of droplet movement due to ultrasonic scaling procedures are shown in Figs. 14 and 15. Figure 14 shows only droplets larger than 25 *μ*m, colored by droplet diameter, whereas Fig. 15 depicts droplets under 25 *μ*m. This distinction in droplet size allows for the separate analyses of large droplets and small droplets, and their differing behavior in the clinic. Although droplets under 100 *μ*m are commonly accepted to be aerosols, substantially differing behavior is observed for droplets above and below the 25 *μ*m threshold in the dentistry clinic. In general, we observe that droplets over 25 *μ*m (Fig. 14) remain isolated within the patient cubicle while droplets under 25 *μ*m (Fig. 15) disperse throughout the clinic.

#### 1. Behavior of droplets over 25 μm in diameter

On the west side of the room, a majority of droplets over 25 *μ*m land near the center of the dental chair, which corresponds to the location of the dental bib on the patient's chest. However, large droplets in cubicles located directly under downward traveling detached jets, such as chairs R2C2 and R3C2, travel transversely and land on the clinic floor. On average, the large droplets on the west side of the room travel less than 1.1 m from the source, as shown in Table VI. Contrarily, the large droplets on the east side of the room exhibit increased advection due to the increased mass flow rate of the vents compared to the west side of the room. On the east side of the clinic, a majority of large droplets travel away from the dental chair, either impacting the floor or cubicle dividers. Specifically, chair R2C6 falls in a region where four jet zones meet, producing a strong downward detached jet into the cubicle immediately east of the patient. The large droplets of R2C6 initially travel upwards after leaving the injection site, before traveling toward, and impacting, the cubicle wall. The large droplets on the east side of the room may travel up to 1.89 m, on average, from the ultrasonic scaling source (Table VI). Ultimately, large droplets from the east-side of the room travel 10%–18% farther on average than those on the west side of the room due to the higher mass flow rates from the vents, increased vorticity, and increased TKE.

**TABLE VI.** Average distance traveled (m) by droplets as a function of their diameter.

Chair	10 *μ*m	20 *μ*m	30 *μ*m	40 *μ*m	50 *μ*m	60 *μ*m	70 *μ*m	80 *μ*m	90 *μ*m	100 *μ*m
R1C1	20.224 9	0.920 3	0.735 0	0.694 9	0.656 7	0.630 1	0.609 5	0.590 5	0.587 0	0.573 7
R1C2	25.878 3	3.472 2	0.775 6	0.727 3	0.677 3	0.636 7	0.605 6	0.583 7	0.568 3	0.559 7
R1C3	23.820 9	0.798 5	0.689 6	0.655 9	0.621 4	0.600 1	0.584 7	0.571 2	0.562 6	0.555 9
R1C4	17.589 5	3.480 0	1.094 1	0.981 2	0.865 6	0.768 6	0.698 5	0.653 6	0.636 6	0.621 8
R1C5	25.566 9	6.268 0	0.674 0	0.666 9	0.648 7	0.593 1	0.573 9	0.537 2	0.527 3	0.525 9
R1C6	28.215 2	10.984 2	0.890 8	0.820 9	0.751 1	0.703 7	0.638 6	0.555 5	0.532 2	0.530 7
R1C7	37.222 2	8.185 7	0.779 9	0.737 0	0.695 1	0.660 4	0.633 0	0.610 3	0.603 1	0.587 8
R1C8	28.892 2	7.173 2	0.828 1	0.781 0	0.724 8	0.678 8	0.650 8	0.620 4	0.606 4	0.594 3
R1C9	25.440 5	6.901 5	0.798 7	0.756 8	0.706 6	0.671 7	0.643 8	0.621 1	0.607 6	0.599 0
R2C1	16.708 4	3.389 4	0.796 0	0.737 1	0.689 3	0.652 6	0.624 4	0.598 6	0.587 4	0.579 4
R2C2	13.750 9	1.496 5	0.903 3	0.819 9	0.740 7	0.679 0	0.632 9	0.600 7	0.586 0	0.576 1
R2C3	20.357 8	0.914 2	0.745 7	0.700 2	0.665 5	0.639 1	0.618 6	0.602 6	0.599 1	0.588 0
R2C4	20.446 2	1.490 8	0.811 8	0.742 7	0.702 9	0.666 2	0.642 0	0.624 7	0.617 7	0.607 1
R2C5	21.348 9	8.205 1	1.445 8	1.352 7	1.231 2	1.086 9	0.954 6	0.878 7	0.800 9	0.735 2
R2C6	35.071 6	19.405 9	1.060 0	1.055 3	0.971 0	0.797 3	0.758 1	0.638 9	0.655 5	0.616 7
R2C7	29.822 1	10.210 7	0.749 4	0.743 9	0.700 6	0.660 6	0.636 3	0.603 7	0.554 8	0.562 6
R2C8	17.385 9	2.050 5	0.726 3	0.681 0	0.642 5	0.612 7	0.590 2	0.578 3	0.559 7	0.552 0
R3C1	11.454 9	0.990 3	0.672 2	0.641 4	0.609 0	0.589 1	0.574 3	0.558 6	0.557 4	0.549 2
R3C2	10.138 4	2.350 3	0.990 1	0.916 8	0.833 1	0.746 4	0.677 4	0.636 5	0.621 2	0.608 5
R3C3	19.094 0	1.816 7	0.699 4	0.670 1	0.640 0	0.619 7	0.603 3	0.590 8	0.583 7	0.576 4
R3C4	15.740 9	1.917 7	0.931 4	0.848 4	0.761 7	0.696 6	0.654 5	0.627 0	0.616 9	0.604 6
R3C5	15.271 3	1.181 3	0.757 0	0.711 6	0.675 3	0.649 5	0.632 4	0.614 7	0.612 0	0.603 3
R3C6	24.967 1	4.982 5	0.730 3	0.698 7	0.652 2	0.617 6	0.593 7	0.567 9	0.548 3	0.542 9
R3C7	27.380 0	10.188 7	0.766 1	0.714 7	0.654 9	0.610 2	0.557 2	0.501 5	0.497 7	0.491 5
R3C8	14.279 8	5.531 9	1.888 5	1.721 0	1.624 4	1.537 6	1.468 7	1.417 3	1.377 2	1.332 8

Due to their increased mass and higher inertia, large droplets remain airborne for short periods of time. As seen in Table VII, large droplets from the west side of the room remain airborne for less than 4.812 s on average. However, large droplets on the east side of the room may remain airborne for up to 7.92 s on average, corresponding to an enhancement in residence time of up to 64.5% for 30 *μ*m droplets. The disparity between large west- and east-side droplets grows and then falls with increasing droplet diameter. The 100, 150, and 200 *μ*m droplets residence times increase up to 165.5%, 84.3%, and 11.8%, respectively. Droplets in the 70 *μ*m–140 *μ*m range are most affected, where their residence times on the east side of the clinic are more than double that of those on the west side. Upon further investigation, this behavior was isolated to the aforementioned fate of large drops on the east side of the room. The 70 *μ*m–140 *μ*m droplets on the east side are more likely to impact the floor or cubicle wall than the patient or dentistry chair, and therefore remain airborne for longer periods of time.

**TABLE VII.** Average residence times of droplets for each injection location in the clinic as a function of droplet diameter. Times are stated in minutes.

Chair	10 *μ*m	20 *μ*m	30 *μ*m	40 *μ*m	50 *μ*m	60 *μ*m	70 *μ*m	80 *μ*m	90 *μ*m	100 *μ*m
R1C1	4.167 6	0.077 2	0.037 5	0.033 1	0.028 4	0.024 9	0.022 2	0.019 6	0.017 1	0.015 7
R1C2	4.357 8	0.537 5	0.052 8	0.044 5	0.037 8	0.032 0	0.027 2	0.023 2	0.019 7	0.018 2
R1C3	6.327 5	0.050 7	0.031 4	0.028 1	0.024 5	0.022 0	0.019 9	0.018 1	0.015 7	0.014 3
R1C4	3.434 3	0.481 9	0.072 8	0.059 0	0.046 3	0.036 5	0.029 1	0.023 5	0.021 5	0.019 5
R1C5	4.555 0	1.419 5	0.048 5	0.044 1	0.039 7	0.031 7	0.027 3	0.022 0	0.019 4	0.017 7
R1C6	4.044 9	1.362 0	0.070 7	0.061 6	0.050 5	0.042 8	0.034 2	0.025 1	0.021 5	0.019 7
R1C7	5.036 8	0.957 1	0.050 1	0.043 8	0.037 7	0.032 3	0.028 0	0.024 0	0.020 7	0.018 9
R1C8	3.763 9	0.966 5	0.056 4	0.049 7	0.041 8	0.035 1	0.030 4	0.025 0	0.021 3	0.019 5
R1C9	4.603 2	0.935 1	0.053 3	0.046 1	0.037 7	0.031 9	0.027 0	0.022 2	0.019 8	0.018 1
R2C1	2.847 3	1.420 3	0.057 7	0.049 1	0.041 4	0.034 8	0.029 3	0.023 6	0.021 5	0.019 4
R2C2	2.424 4	0.187 9	0.062 6	0.052 0	0.042 3	0.034 6	0.028 6	0.023 4	0.020 6	0.019 0
R2C3	3.804 0	0.077 0	0.040 8	0.035 3	0.030 7	0.026 9	0.023 8	0.020 9	0.018 2	0.016 8
R2C4	3.823 4	0.299 3	0.049 8	0.040 0	0.034 4	0.028 9	0.025 1	0.021 7	0.018 9	0.017 5
R2C5	3.953 4	1.324 4	0.111 3	0.094 3	0.077 6	0.061 9	0.048 7	0.040 5	0.033 4	0.027 6
R2C6	4.866 3	2.402 4	0.092 2	0.083 3	0.071 7	0.052 2	0.043 9	0.032 0	0.030 0	0.025 4
R2C7	3.615 8	1.239 7	0.059 0	0.054 0	0.046 7	0.039 7	0.034 3	0.028 7	0.022 1	0.020 6
R2C8	2.357 9	0.275 2	0.048 3	0.042 3	0.036 5	0.031 2	0.027 1	0.023 9	0.020 0	0.017 8
R3C1	4.167 6	0.077 2	0.037 5	0.033 1	0.028 4	0.024 9	0.022 2	0.019 6	0.017 1	0.015 7
R3C2	2.486 4	0.442 1	0.080 2	0.066 5	0.053 2	0.041 5	0.032 1	0.025 3	0.022 8	0.020 4
R3C3	7.312 2	0.291 8	0.033 5	0.030 2	0.026 7	0.023 9	0.021 5	0.019 5	0.016 9	0.015 6
R3C4	3.349 2	0.291 6	0.062 7	0.052 2	0.041 6	0.034 1	0.028 6	0.023 8	0.021 5	0.019 9
R3C5	2.551 8	0.141 9	0.037 9	0.032 7	0.028 6	0.025 4	0.022 8	0.020 2	0.017 8	0.016 6
R3C6	4.501 7	0.686 2	0.047 2	0.041 9	0.035 7	0.030 5	0.026 5	0.022 8	0.018 7	0.017 1
R3C7	3.999 8	1.540 6	0.065 8	0.057 0	0.045 9	0.037 4	0.029 5	0.021 8	0.019 4	0.017 4
R3C8	2.756 9	0.771 4	0.132 0	0.108 8	0.094 3	0.082 2	0.072 8	0.065 7	0.059 8	0.054 3

Per the simulation results, all large droplets (>25 *μ*m) impact and contaminate surfaces in the dental clinic. No large droplets in the simulation were found to escape through the exhaust vents of the room. The data highlights the necessity to adequately disinfect surfaces surrounding the patients in the clinic to prevent the potential spread of disease. This result also shows that filtration on either the intake or exhaust vents within the clinic would have no effect on droplets in this size range. A more detailed discussion of surface contamination based on droplet fates follows in Sec. V B 4, and includes large and small droplets.

#### 2. Behavior of droplets under 25 μm in diameter

Figure 15 illustrates the wide-spread of small droplets throughout the dental clinic when considering all droplets and aerosols generated by procedures occurring in all patient cubicles. The droplets are colored by diameter. As shown in the figure, the entire clinic may be saturated by droplets during the performance of ultrasonic dental scaling procedures. Although no droplets smaller than 20 *μ*m are injected in the simulation, the evaporation of the droplets' volatile fraction allows them to reduce in size to 6.4 *μ*m diameter (Sec. IV B 2). The aerosolized droplet nuclei of less than 20 *μ*m are the remnants of droplets as large as 62.5 *μ*m and consist of saliva, impurities in the ultrasonic scaler's irrigant water supply, and virions. The droplet behavior in this size range is discussed in detail since prior studies have identified droplets ranging from 10 *μ*m to approximately 50 *μ*m pre-evaporation diameter as having the highest infection probability (Chaudhuri *et al.*, 2020).

The salient details of average droplet residence time and average distances traveled are presented in Tables VI and VII. The aerosol droplet nuclei remain airborne for up to 7.31 (R3C3) and 2.4 (R2C6) min on average for 10 and 20 *μ*m droplets, respectively. During that time, the aerosol nuclei travel up to 19.09 m (R3C3) and 19.41 m (R2C6) on average for 10 and 20 *μ*m droplets, respectively. Similarly, the 10 *μ*m aerosol nuclei of R1C7 average 37.22 m in 5.04 min. This result is of particular concern due to the fact that the aerosols are capable of traversing half of the perimeter of the room on average, meaning that many droplets may travel substantially farther. However, there exists a disparity between the dentistry clinic's two sides, as was observed with the large (>20 *μ*m) droplets. On average, the aerosols on the west side of the room remain airborne 4.04 and 0.35 min for 10 and 20 *μ*m droplets, respectively; however, the 10 and 20 *μ*m droplets on the east side of the room reach their fate within 3.83 and 1.09 min, respectively. We observe that the 10 *μ*m droplets' residence time does not change substantially in the east side of the room, where there the vents have increased mass flow rates. However, the 20 *μ*m droplets' residence time increases 3.1 times on the east side of the clinic when compared to the west side.

Regarding distance traveled, the west side of the room averages 17.93 m and 1.92 m for 10 and 20 *μ*m droplets, respectively. However, on the east side of the room 10 and 20 *μ*m droplets travel an average of 25.45 m and 7.86 m, respectively. The results indicate that although 10 *μ*m aerosol residence time differs little between the two sides of the room, those on the east side travel 42% farther on average due to their increased velocity. The 20 *μ*m droplet travel distances are significantly increased on the east side of the room, nearly 4.1 times those at the west side of the room, signifying that droplets in this diameter range are of particular concern with respect to flow conditions within the clinic.

**FIG. 16.**
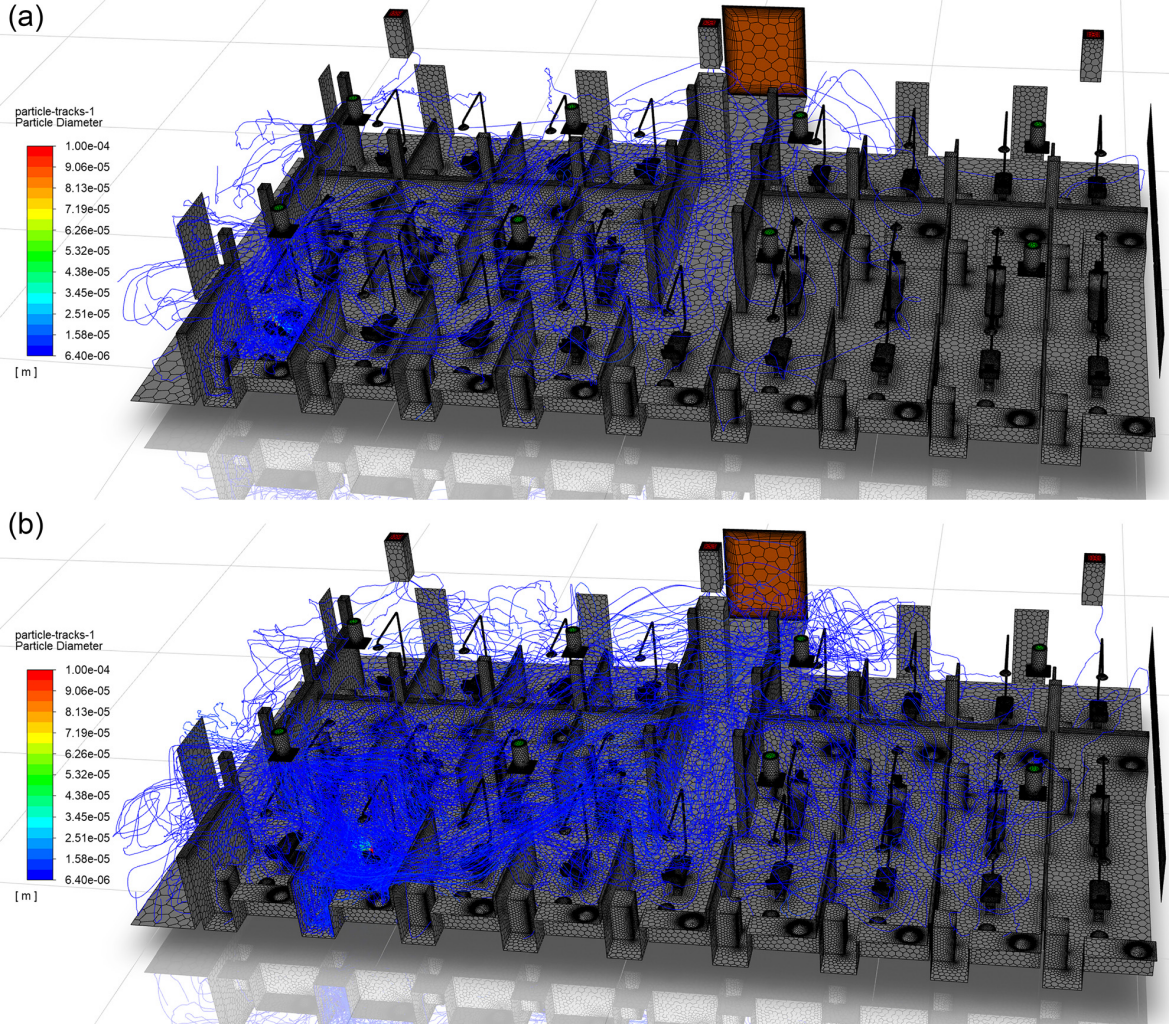
Comparison of the droplet distribution in the clinic resulting from ultrasonic scaling procedures performed in chairs (a) R1C1 and (b) R1C2.

**FIG. 17.**
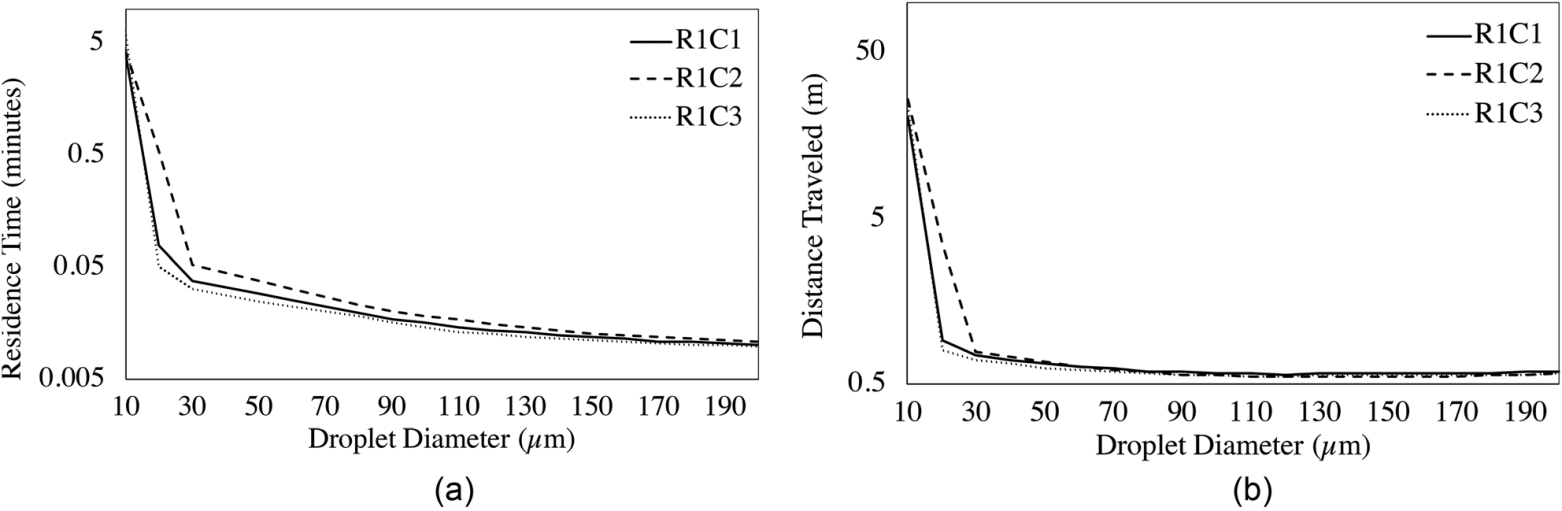
The average (a) residence time and (b) distance traveled as a function of droplet diameter for chairs R1C1, R1C2, and R1C3.

A large factor influencing small droplet behavior (<20 *μ*m) is the entrainment of droplets in the detached jets from the ceiling vents. Patient chairs that reside directly beside one another may exhibit substantially differing droplet behavior. Figure 16 depicts patient chairs R1C1 and R1C2, where the droplet tracks are colored by diameter. From a qualitative analysis, the droplets generated in R1C2 are observed to travel farther and spread wider throughout the clinic than those originating in R1C1. The primary difference between the two chairs is the presence of a detached jet entering R1C2 (Fig. 10) immediately east of the patient chair. The small droplets are entrained in the jet, follow the streamlines in this region, and expel a large number of droplets from the patient cubicle. The droplets are then observed to spread throughout the clinic following the streamlines originating from vent South 4 (pink streamlines in Fig. 12). The interaction of droplets with the detached jet substantially increases the residence times and travel distances of aerosols. The droplets originating in R1C1 and R1C3 follow a similar trend in residence time, as seen in Fig. 17(a), due to the absence of a detached jet in these cubicles. However, R1C2 has increased residence times for droplets below 150 *μ*m, with the largest enhancement in time occurring in the 20 *μ*m range. Similar behavior is observed in the droplets' distances traveled. Figure 17(b) shows that R1C1 and R1C3 behave similarly in droplet distance traveled for patient cubicles that do not contain detached jets. However, the 20 *μ*m range droplets travel substantially farther due to the detached jet's presence in R1C2. The trend of increased droplet residence time and travel distance due to the presence of detached jets in cubicles (Figs. 11 and 12) can be observed in the data presented in Tables VI and VII.

The worst-case scenarios for droplets cause significant concern with regard to residence time and distance traveled. The previous discussion focused on mean values; however, individual droplets may remain airborne for long durations of time. The most concerning patient treatment location in the dentistry clinic is R2C6 due to its location under a detached jet formed by the interaction of four vents and relatively high TKE. Ultrasonic scaling procedures performed in R2C6 can produce 20 *μ*m droplets that rapidly evaporate to 6.4 *μ*m aerosols and travel up to 2026.7 m in 3.35 days before being exhausted through the clinic's ventilation. Similarly, a limited number of evaporated droplet nuclei in the 20 *μ*m range may travel up to 319.52 m in 8.93 h prior to settling on a surface in the clinic. Droplets and nuclei sized 30 *μ*m and above travel no more than 39.64 m in 4.85 min in the worst-case scenario. Although the results assume steady-state conditions with no movement in the clinic or change in flow conditions, the data shows the need to ventilate enclosed spaces with fresh air where aerosol-generating procedures are performed.

The droplets in the clinic exhibit differing fates based on local flow conditions. Previously, in Sec. V B 1, large droplets were observed to either land on the floor, patient, or cubicle walls. However, small droplets are capable of escaping through the room's ventilation. Per the simulation results in Table VIII, only 1.646% of the total droplets from patient treatment areas escape through exhaust ventilation. The quantity of escaped droplets varies based on the patient cubicle location relative to ventilation locations. As shown in Table VIII, as little as 0.314% of the droplets escape through ventilation in R1C3, which is a cubicle located far from the exhaust vents and does not contain a detached jet. Contrarily, as many as 3.953% of droplets generated in R3C8 escape through exhaust ventilation due to being located directly below an exhaust vent and containing a detached jet from the east wall. Per these data, few droplets enter the heating, ventilation, and air conditioning (HVAC) system and a vast majority (98.354%) land on surfaces within the clinic, causing potentially dangerous surface contamination (He *et al.*, 2021). These results fall in line with current CDC recommendations since few droplets exit through the exhaust vents from the clinic. The CDC does not provide guidance on decontamination of HVAC systems potentially exposed to SARS-CoV-2 since there has been no evidence demonstrating risks associated with viable virus contamination (Centers for Disease Control and Prevention, 2020a).

**TABLE VIII.** Droplet fate based on source location. Droplets either escape through the exhaust ventilation, or land on a surface within the clinic.

Source location	Escape through vents (%)	Land on surface (%)
R1C1	0.428	99.572
R1C2	0.924	99.076
R1C3	0.314	99.686
R1C4	0.814	99.186
R1C5	0.986	99.014
R1C6	1.062	98.938
R1C7	0.929	99.071
R1C8	1.529	98.471
R1C9	3.175	96.825
R2C1	2.856	97.144
R2C2	1.810	98.190
R2C3	0.836	99.164
R2C4	1.041	98.959
R2C5	1.376	98.624
R2C6	1.890	98.110
R2C7	1.688	98.312
R2C8	2.055	97.945
R3C1	1.586	98.414
R3C2	3.822	96.178
R3C3	1.632	98.368
R3C4	1.954	98.046
R3C5	1.074	98.926
R3C6	1.125	98.875
R3C7	2.310	97.690
R3C8	3.953	96.047

#### 3. Surface contamination in the clinic due to droplet settling

Although COVID-19 primarily spreads through close contact and airborne aerosols, transmission through direct contact with contaminated surfaces is possible (Centers for Disease Control and Prevention, 2020a; He *et al.*, 2021). Recent literature suggests SARS-CoV-2 may remain viable on different surfaces under varying temperature and humidity levels (Chin *et al.*, 2020; van Doremalen *et al.*, 2020). Prior publications of the CDC infection prevention checklist for dental settings (Centers for Disease Control and Prevention, 2020b) heavily emphasized sterilization and disinfection of patient-care items and devices, however, had limited elements assessed regarding environmental infection prevention and control. The environmental elements addressed were limited to routine cleaning and disinfection of surfaces. The CDC recommendation included the cleaning of countertops and dental units with a low- or intermediate-level disinfectant between patient visits, or to barrier-protect surfaces and disinfect them at the end of the day. General housekeeping surfaces, such as walls and floors, were recommended to be cleaned using a low-level disinfectant with detergent on a regular basis, when spills occur, or when the surface had visible soiling. The housekeeping surfaces were noted as having the limited risk of disease transmission, and therefore could be decontaminated less rigorously than patient-care items (Centers for Disease Control and Prevention, 2003). During the COVID-19 pandemic, the CDC updated its environmental infection control recommendations to include routine cleaning and disinfection procedures for frequently touched surfaces and patient-care areas where aerosol-generating procedures are performed (Centers for Disease Control and Prevention, 2020a). However, the current simulation results show that surface contamination can occur outside the patient-care area due to droplet and aerosol deposition.

**FIG. 18.**
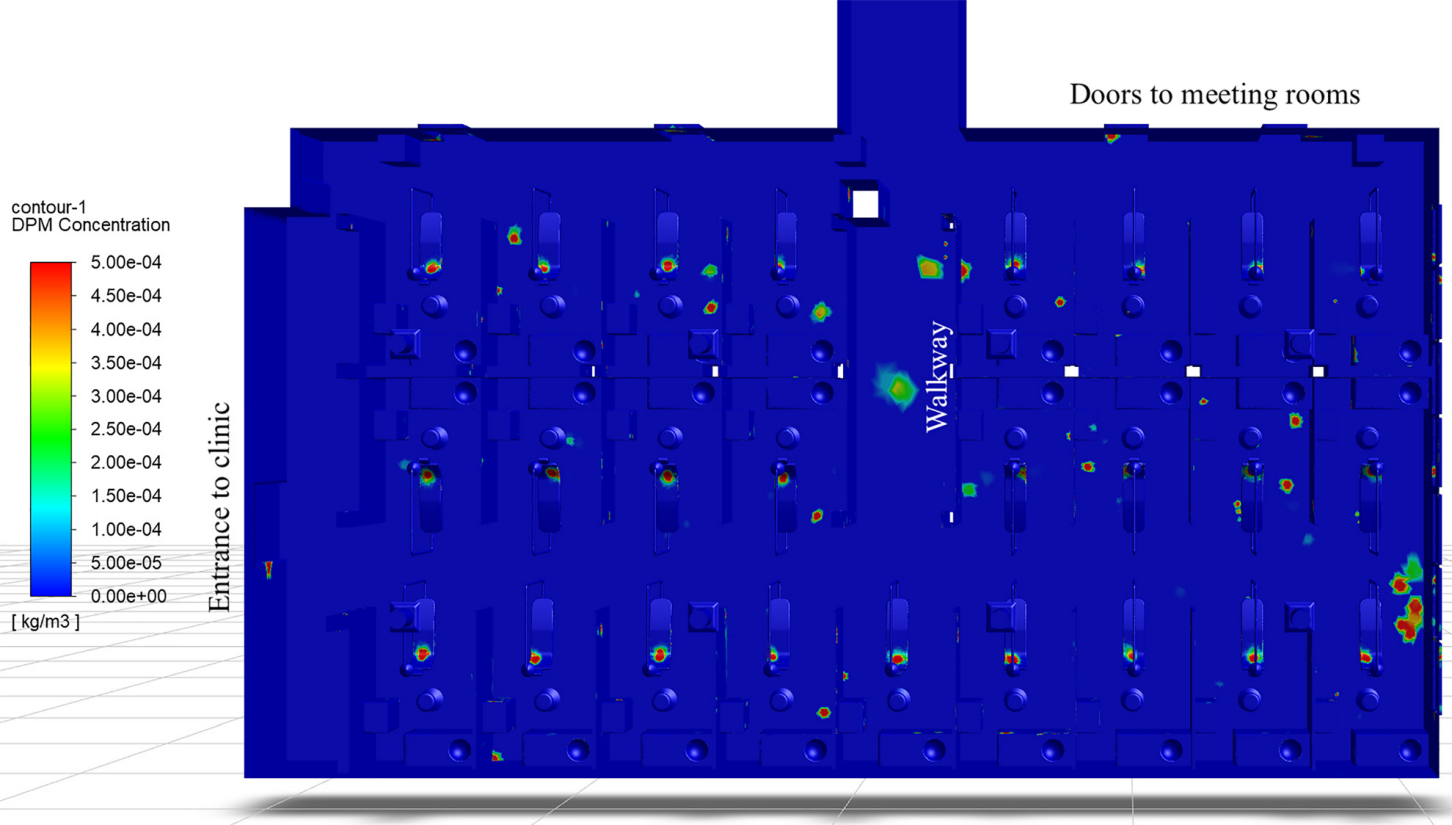
Surface concentration of droplet mass in the dentistry clinic as viewed from above.

Figure 18 depicts the surface concentrations of droplet mass in the clinical dentistry setting. The high concentration regions correspond to locations where droplets settle and potentially contaminate the surface. Much of the contamination occurs on the dental chair, which would correspond to the location of the dental bib on the patient's chest during an ultrasonic scaling procedure. The simulation results agree with the recent experimental findings of Kaufmann *et al.* (2020), where the authors found ultrasonic scaling results in the highest contamination on the patient's chest, patient's forehead, and the practitioner's gloves. In Fig. 18, there exist many contaminated areas outside of the immediate treatment area. Areas of contamination include the sink countertops, the bowl of the sink itself, and the utility cart beside the patient. These findings also agree with experimental measurements, where the highest contamination was found near the patient, and areas of decreasing contamination were found away from the patient (Kaufmann *et al.*, 2020). However, the aforementioned locations correspond to the updated CDC recommendations for cleaning frequently touched surfaces and patient-care areas. Particular areas of interest that are not included in the CDC recommendations for increased cleaning/disinfection include the floors of R1C4–5, R1C9, R2C2, R2C4–9, and R3C2–5. Particularly, the region of the floor between R1C9 and R2C8 shows a large quantity of deposited droplets. There also exist areas entirely outside the patient treatment cubicles where droplets land and contaminate surfaces. Two concerning locations within the clinic are the floor of the walkway between R3C4 and R3C5, and the floor directly outside the second meeting room in the northeast corner of the clinic. Additional surface contamination is observed near the entrance doorway on the west side of the clinic. Finally, droplets also contaminate the dividers between treatment cubicles. These locations are of interest since the CDC classifies them as general housekeeping surfaces, which are not decontaminated as rigorously as patient treatment areas. In the context of the simulation results, the authors would recommend treating these areas similar to other high-risk surfaces in the clinic and following disinfection procedures akin to patient-care areas, to prevent possible infection due to contact with the contaminated surfaces.

#### 4. Airborne aerosol concentration

Airborne aerosol concentration is important in determining the risk that patients and practitioners experience in the dental clinic. Inhalation of airborne aerosols, droplets, or droplet nuclei that contain viable SARS-CoV-2 virions poses an infection risk. This risk is of heightened concern due to the recent results of Lednicky *et al.* (2020) demonstrating that aerosols can transport viable SARS-CoV-2 virus up to 4.8 m in the absence of aerosol-generating procedures. The transport of viable viruses may be increased in clinical dentistry settings due to the presence of aerosol-generating procedures, such as ultrasonic scaling or the use of high-speed drills.

**FIG. 19.**
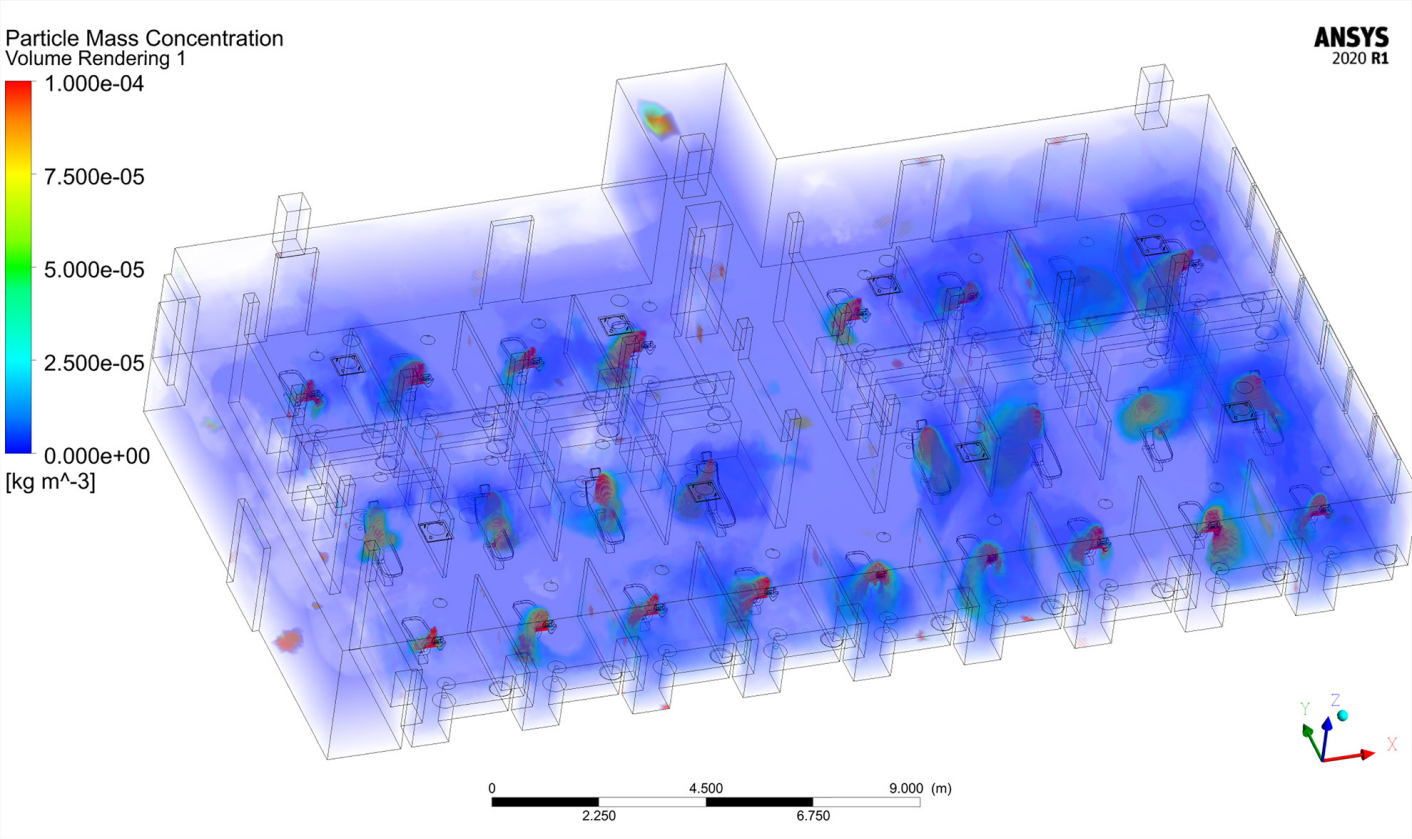
Volume rendering of airborne droplet concentration in the dentistry clinic simulation.

Figure 19 shows a volume rendering of airborne droplet mass concentration from ultrasonic scaling procedures performed in all patient treatment areas. The highest concentrations, located near the head of the patient, arise due to large droplets ejected from the patient's mouth during the procedure. There exist few locations within the clinic setting where there are no aerosols present, meaning that the entirety of the dental clinic may pose a risk for infection due to inhalation. Higher aerosol concentrations on the west side of the clinic reside in treatment areas with increased TKE (Fig. 13) and lower vorticity (Fig. 11), such as R2C2, R2C4, R3C2, and R3C4, compared to their surroundings. This result agrees with prior studies on the behavior of inertial particles. Inertial particles are expelled from high-vorticity regions and concentrate in high-strain regions (Maxey, 1987; Squires and Eaton, 1991; and Eaton and Fessler, 1994). Similarly, preferential clustering or accumulation of aerosols occurs in high-strain, low-vorticity regions (Squires and Eaton, 1991; Chun *et al.*, 2005). The east side of the room exhibits higher local concentrations due to increased residence times of droplets as well as increased TKE compared to the west side of the room.

The concentrations of droplet mass in the patient treatment areas may appear low, on the order of $10^{-5}$ kg/m^3^; however, the viral load may be high. To the authors' best knowledge at this time, there have been no studies of viral load in aerosolized droplets from dentistry procedures. However, an estimate may be provided by utilizing the median SARS-CoV-2 viral load of saliva specimens by To *et al.* (2020) of $3.3\times 10^{6}$ copies/ml. Under the assumption that the irrigant water from ultrasonic scaling does not dilute the saliva and evaporation does not affect viral load, we find that the region immediately near the patient could contain upwards of $3.3\times 10^{5}$ copies/m^3^ of virus in the air. Regions away from the patient in low-concentration treatment areas may contain $2\times 10^{4}$ copies/m^3^. Assuming a normal inhalation tidal volume of 500 ml (approximately 7 ml/kg body mass), an individual may inhale up to 165 copies of virus immediately near the infected patient being treated, or 15 copies far from the patient in a non-treatment area, per breath. These estimates may be improved by future studies examining the viral load of evaporating droplets generated by dental procedures. The current estimates would be considered a worst-case scenario since it is expected the irrigant water from the ultrasonic dental scaler would dilute the human saliva, resulting in a lower viral load. Furthermore, a study that establishes the number of SARS-CoV-2 virus copies required to infect a healthy human is necessary to exactly determine the risk in different areas of the clinic.

## VI. CONCLUSIONS

Access to medical and dental procedures is a basic human necessity, even amidst ongoing or future global pandemics. However, the generation of virus-laden droplets, or aerosols, in dental procedures can pose substantial risks to patients and healthcare workers alike. In this research, we used computational fluid dynamics to quantify the transport of large droplets and aerosols in clinical dentistry settings to understand the risks associated with a common dental procedure, ultrasonic scaling. The data produced from these simulations may be used to improve policies regarding procedures during outbreaks of human contagions or pandemics. Several conclusions may be drawn from the data presented in this study, as follows:

• The locations of vents relative to the patient undergoing an aerosol-generating procedure greatly determine the spread of potentially virulent droplets and aerosols in a clinical setting.
• An imbalance in the ventilation system (i.e., different vents flowing at substantially different rates) can create intense turbulence and a disparity in the behavior of the droplets in different areas of the room.
• Aerosols below 15 *μ*m remain airborne for up to 7.13 min on average; however, a small quantity of droplets may remain airborne for days without proper ventilation.
• Aerosols below 15 *μ*m can travel up to 25.45 m on average from their source, potentially contaminating entire clinics.
• The distance traveled by large droplets of over 60 *μ*m averages below the six-foot social distancing guidelines set forth by the CDC. However, large droplets pose a surface contamination risk inside the patient cubicles.
• Decontamination and sterilization efforts should be extended to include all possible surfaces of the clinic that a patient or practitioner may contact.

The ideal solution to prevent the airborne transmission of disease in medical settings is to eliminate the generation of virus-laden droplets and aerosols. This assessment falls in line with current CDC guidelines, such as wearing a mask to minimize airborne droplets generated by coughing, sneezing, speaking, and breathing. However, wearing a mask is not conducive to dental procedures, such as ultrasonic scaling. Additionally, local suction is insufficient to capture all droplets generated by such procedures.

There exist two possible solutions that may be implemented in dental clinics to prevent or significantly decrease the generation of airborne droplets from scaling procedures. The first solution is to halt the use of ultrasonic scaling devices and revert to the use of mechanical dental scalers and picks, which do not use irrigant water flow and therefore do not generate aerosols. The second possible solution is the addition of high molecular weight polymer additives to the irrigant water supply, which substantially reduces or eliminates aerosol generation. The recent work of Plog *et al.* (2020) demonstrated that the use of Food and Drug Administration (FDA)-approved additives to irrigation solutions prevented droplet formation through the introduction of viscoelastic forces. The additives were shown to work when used in both ultrasonic dental scalers as well as high-speed dental drills (not considered in this study) (Plog *et al.*, 2020). Other possible solutions that may be further examined are novel high-speed evacuation devices that capture most, or all, droplets generated during these procedures. For example, Jia *et al.* (2021) have recently proposed a vacuum helmet to enclose the patient's head, while allowing access to the patient's mouth. Their simulations show that such a device may substantially reduce the expulsion of aerosols.

The data presented in this research can be used in conjunction with future studies to establish aerosol exposure guidelines in clinical dentistry settings. However, to the best knowledge of the authors, two critical pieces of information are required to establish such guidelines that are currently not available in the literature. The first necessary information is a study determining the SARS-CoV-2 virion concentration in droplets and aerosols expelled during dental procedures. Such data would allow the airborne aerosol concentration (Fig. 19) to be directly related to airborne viral load. Second, data regarding the infectivity of SARS-CoV-2 is required to relate the airborne virion concentration to the risks presented in different areas of the clinic. Specifically, a study characterizing the mean infection risk when exposed to certain quantities of virions, or genomic copies, is required. With these two additional pieces of information, the data in Fig. 19 could be represented as infection risk based on the patient or practitioner location in the clinic.

## Data Availability

The data that support the findings of this study are available from the corresponding author upon reasonable request.
